# Diversity of Fungal Communities on Diseased and Healthy Cinnamomum burmannii Fruits and Antibacterial Activity of Secondary Metabolites

**DOI:** 10.1128/spectrum.00080-23

**Published:** 2023-05-10

**Authors:** Wei Wang, Teng Cai, Yuting Yang, Hui Guo, Zhuo Shang, Hamza Shahid, Yirong Zhang, Sirun Qiu, Xinnian Zeng, Xiaoli Xu, Yi Liu, Ping Fang, Ping Ding, Ziling Mao, Tijiang Shan

**Affiliations:** a College of Forestry and Landscape Architecture, South China Agricultural University, Guangzhou, China; b College of Plant Protection, South China Agricultural University, Guangzhou, China; c School of Pharmacy, Tongji Medical College, Huazhong University of Science and Technology, Wuhan, China; d College of Pharmaceutical Sciences, Zhejiang University of Technology, Hangzhou, China; e School of Pharmaceutical Sciences, Shandong University, Jinan, China; f Instrumental Analysis and Research Center of SCAU, South China Agricultural University, Guangzhou, China; g School of Pharmaceutical Sciences, Guangzhou University of Chinese Medicine, Guangzhou, China; Agroscope

**Keywords:** fungal communities, endophytic fungi, *Cinnamomum burmannii*, secondary metabolites, antibacterial activity

## Abstract

The composition and structure of fungal communities on healthy and diseased fruits of Cinnamomum burmannii (Nees and Nees) Blume were characterized, with evaluation of the antibacterial activity of secondary metabolites from culturable fungi following the first identification of secondary metabolites in the fungus Medicopsis romeroi (Esf-14; GenBank accession number OK242756). These results are significant for understanding the functional variation in bioactivity in fungal communities and developing a broader range of bioactive resources. High-throughput sequencing results indicated that the fungal community in diseased fruit differed from that in healthy fruit at the phylum, class, order, or genus level, with significant differences in the species and relative abundance of the dominant flora. A total of 49 (healthy fruit) and 122 (diseased fruit) artificially cultivable endophytic fungi were isolated, and 41 different strains (11 from healthy fruit and 30 from diseased fruit) were successfully identified by morphological and molecular biological analyses, which were classified into 8 groups and 23 genera by phylogenetic tree analysis, with Pleosporales, Glomerellales, and Hypocreales being the dominant groups at the order level and *Colletotrichum* being the dominant group at the genus level. The results of the antibacterial assay demonstrated that the secondary metabolites of all strains had different degrees of antibacterial activity, while the secondary metabolites of endophytic fungi from diseased fruit were generally stronger than those of fungi from healthy fruit, with the active secondary metabolites dominated by small and moderately polar compounds. Combined analysis of fungal communities, phylogenetic tree analysis, and bioactivity analysis of culturable strains revealed strong antibacterial activity of both upregulated and downregulated flora in diseased fruit. Five compounds, including two new (5,6-dimethoxy-[1′,1:4,1″-terphenyl]-2-ol [compound 1] and 5-(methoxycarbonyl)-2-methylbenzo[d][1,3]dioxole-2-carboxylic acid [compound 2]) and three known compounds (3,7-dihydroxy-1,9-dimethyldibenzofuran [compound 3], methyl 3-hydroxybenzoate [compound 4], and uracil [compound 5]), were isolated and identified for the first time from the endophytic fungus *Medicopsis romeroi*. In general, the diversity of fungal communities on diseased fruit was lower than that on healthy fruits, while the antibacterial activity of artificially cultured endophytic fungi on diseased fruits was generally stronger than that on healthy fruits, suggesting excellent promise for the development of secondary metabolites from active strains on diseased fruit as antibacterial agents.

**IMPORTANCE** Powdery fruit disease is a notorious disease of *Cinnamomum burmannii* that causes severe loss in fruit production. Studies on the function of endophytic fungal communities in healthy plant tissues are not new, while little is known about the functional changes of fungal communities in disease-causing plant tissues. Our results demonstrate that fungal communities in diseased fruits differ from those in healthy fruits at the level of phylum, class, order, or genus, with significant differences in the species and relative abundance of dominant groups. Endophytic fungi in diseased fruits appeared to produce secondary metabolites with stronger antibacterial properties, although the community diversity was not as varied as that in healthy fruits. In addition, secondary metabolites of the *Medicopsis romeroi* strain from diseased fruits were identified for the first time. These results have important implications for understanding the functional variation of bioactivity in fungal communities and for developing a broader resource of bioactivity.

## INTRODUCTION

Plant secondary metabolites and their derivatives are considered to be important sources of new natural active pharmaceuticals and are essential alternatives to synthetic chemicals (1, 2). However, the yields of secondary metabolites extracted from plants are low, and large quantities of plant materials are needed to obtain larger amounts of chemical components. Therefore, extensive research on these chemicals could cause direct harm to ecosystems (3). Plant endophytic fungi are a novel and underexploited resource that harbor potentially active molecules (4). Endophytic fungi reside in plant tissues without causing disease symptoms and have a significant impact on the host plant, as they can increase plant resistance by producing a wide range of active macromolecules (5–7). Endophytic fungi such as Aspergillus fumigatus, Rhizopycnis vagum, Diaporthe maritima, and Phialophora mustea have been widely reported to produce antimicrobial active compounds (8). In addition, endophytic fungi produce similar biologically active secondary metabolites as the host plant (9). Research on the active secondary metabolites of plant endophytic fungi is essential for the conservation of plant diversity and the environment. However, due to the limitations of endophytic fungal secondary metabolites derived from traditional plant parts, an increasing number of researchers have turned their attention to the identification of promising endophytic microorganisms from specific environments or less widely studied plant parts (10).

Plant characteristics can have a significant impact on the diversity and community composition of endophytic fungi (11, 12). The secretion of active secondary metabolites by endophytic fungi is also altered to facilitate plant responses to biotic or abiotic challenges, such as pathogens, drought conditions, and salinity (13). A previous study showed differences in the endophytic assemblages on yellowing and healthy leaves of citrus plants due to the possible effects of leaf yellowing on endophytic growth (14). This indicates that the relationship between plants and endophytic fungi is not static (15). Cinnamomum burmannii is a small tree of the Lauraceae family called Indonesian cassia, with a wide distribution in Vietnam, Indonesia, Philippines, Myanmar, India, and China, that can be used as a raw material for spices and is also considered a traditional herb in folklore for the treatment of several health disorders (16–18). *C. burmannii* extracts have been demonstrated to possess antioxidant (19), anti-inflammatory (20), antibacterial (21), and anticancer (22) activities. The phytochemical composition of *C. burmannii* is diverse and rich in flavonoids and alkaloids (23). The main constituents reported in the study were cinnamyl alcohol, coumarin, cinnamic acid, cinnamaldehyde, anthocyanin, and essential oils. Previous studies have mostly examined active secondary metabolites from endophytic fungi in healthy plant tissues (24), while the bioactive capacity of endophytic fungal secondary metabolites in diseased tissues has been given little attention. Studies have suggested that while plant tissues are susceptible to invasion by other pathogens after disease, the endophytic flora of plants also undergo important functional changes, such as detoxification, biofilm formation, and activation of microbiome-related signaling pathways (25), implying that endophytic fungi in diseased plants may also be rich in bioactive secondary metabolites. The pathogens of powdery fruit have been discussed in previous studies, and significant differences in the volatile components have been observed between diseased and healthy fruit due to the influence of pathogens (26). However, the diversity of fungal communities and the antibacterial activity of secondary metabolites of culturable fungi in healthy and diseased fruits have not been clearly elucidated.

In this study, the composition and structure of the endophytic fungal communities on healthy and diseased fruits of *C. burmannii* were characterized by high-throughput sequencing. Then, 41 endophytic fungi were isolated and identified from the healthy and diseased fruits, and the antibacterial activity of the secondary metabolites of these endophytic fungi was compared. By combining the results of phylogenetic tree analysis and fungal community characterization, one active strain (*Medicopsis romeroi* Esf-14) was screened, followed by the isolation and identification of two new compounds and three known compounds from this species. The investigation of fungal community variation, culturable endophytic fungal communities, and secondary metabolites from healthy and diseased fruits is important for understanding fungal community function and developing a wider spectrum of bioactive resources.

## RESULTS

### Analysis of Illumina sequencing results.

A total of 212,165 effective sequences were obtained by high-throughput sequencing of six samples of diseased and healthy fruits of *C. burmannii* (see Fig. S2 in the supplemental material). The numbers of operational taxonomic units (OTUs) in the healthy and diseased fruit groups were 114 and 597, respectively, with an average value of 35,361 effective tags. The final effective tags had an average of 8,554,061 bp, with an effective sequence length of 234 to 258 (with an average length of 241.55). The clustering was performed at a 97% similarity level, yielding an average of 90 OTUs. The quality control results showed that the (Sequencing quality control values greater than or equal to 20) Q20 was above 79% and the (Sequencing quality control values greater than or equal to 30) Q30 was above 75%. Rarefaction curves were constructed by randomly selecting sequences and their corresponding diversity indices (see Fig. S3 in the supplemental material). The observed species index and Shannon index dilution curves for both individual and intergroup samples tended to be flat, indicating that the sequencing data were reasonably reliable and accurately reflected the microbial information in the samples.

### Community composition of healthy and diseased fruits.

**FIG 1:**
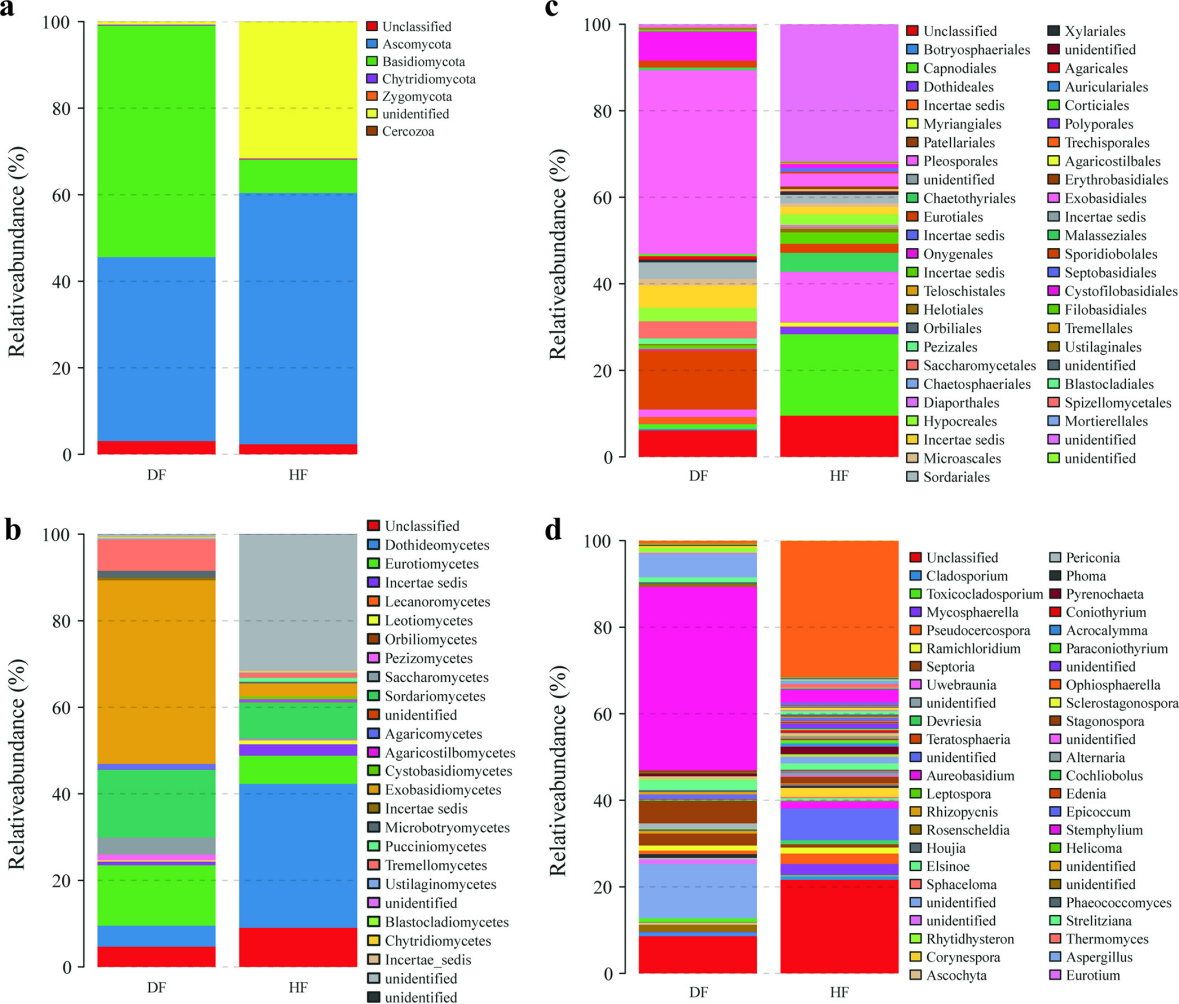
Relative abundance of fungi in healthy and diseased fruit samples of *C. burmannii*. (a) Phylum level. (b) Class level. (c) Order level. (d) Genus level.

Based on the species annotation results, a total of 5 phyla, 19 classes, 39 orders, 57 families, and 116 genera were identified. The relative abundance of species identified at the phylum and genus levels in each sample was calculated based on absolute OTU abundance and annotation information (Fig. 1). The results of the relative abundance plots indicated that the fungal communities in healthy (HF) and diseased fruit (DF) were similar in composition at the phylum level, with Basidiomycota (HF, 7.67%; DF, 53.48%), Ascomycota (HF, 58.10%; DF, 42.51%), and Chytridiomycota (HF, 0.20%; DF, 0.26%) being the dominant groups common to both healthy and diseased fruits; however, the relative abundance of the dominant phylum Basidiomycota (HF, 7.67%; DF, 53.48%) was clearly different. A similar situation was observed at the class level, where Eurotiomycetes (HF, 6.50%; DF, 13.99%) and Sordariomycetes (HF, 8.30%; DF, 15.64%) were the dominant groups common to both healthy and diseased fruits, with Dothideomycetes (33.32%) being the dominant group specific to healthy fruit and Exobasidiomycetes (42.45%) and Exobasidiomycetes (42.45%) being dominant in diseased fruit. At the order level, there were no identical dominant colonies in healthy and diseased fruits, and the relative abundance of dominant colonies in both healthy and diseased fruits was less than 20%, with Capnodiales (18.81%), Pleosporales (11.66%), and Chaetothyriales (4.37%) dominant in healthy fruit and Exobasidiales (42.45%), Eurotiales (13.63%), and Cystofilobasidiales (6.80%) dominant in diseased fruit. Interestingly, at the genus level, *Clinoconidium* (HF, 2.97%; DF, 42.45%) was the dominant colony in both healthy and diseased fruits, while *Aspergillus* (12.57%) and *Guehomyces* (5.30%) were the dominant colonies specific to diseased fruit, while the relative abundance of all of the colonies in healthy fruit was less than 3%.

**FIG 2:**
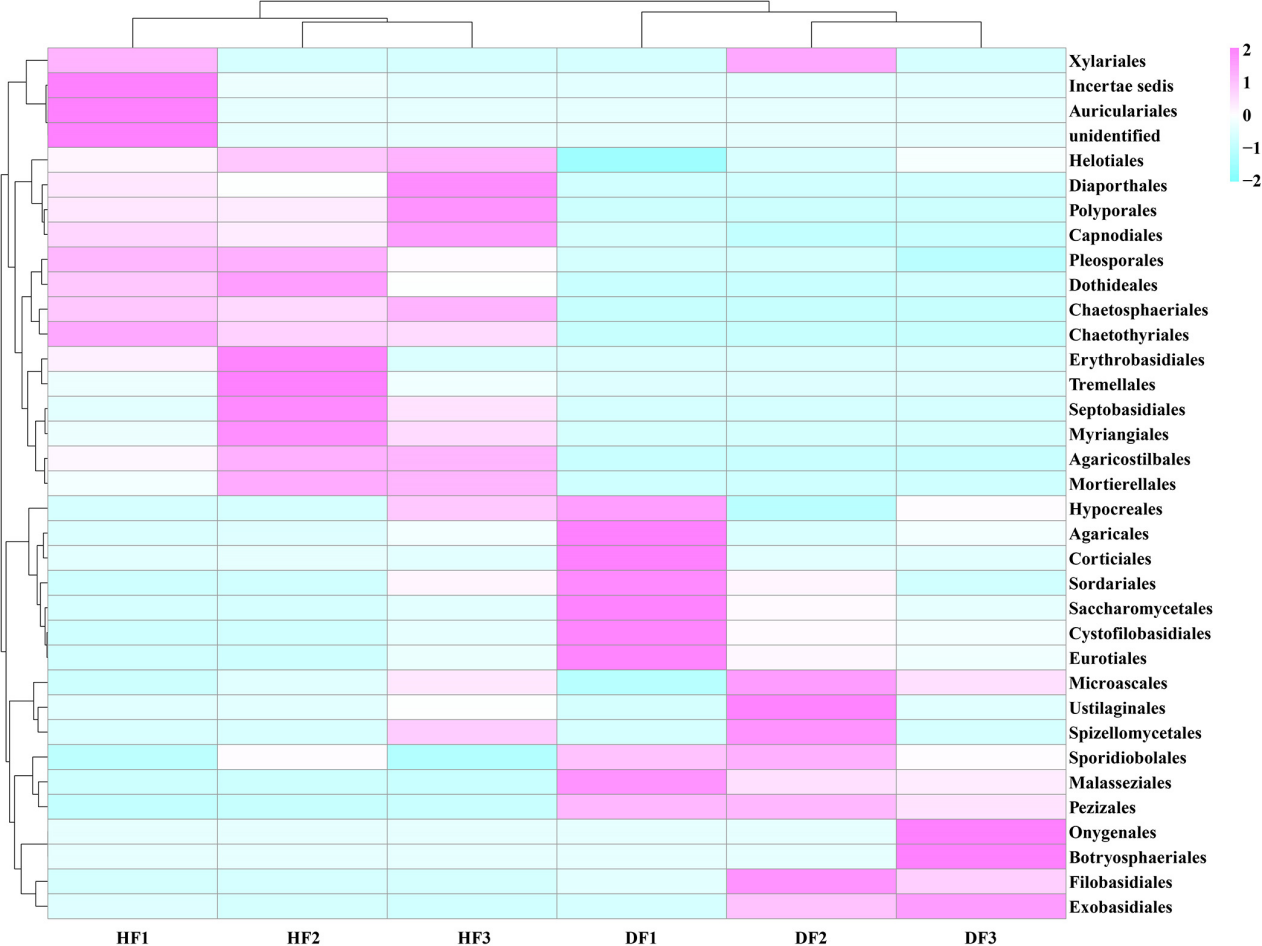
Heatmap of fungi in healthy and diseased fruit of *C. burmannii* at the order level. The value corresponding to the color of each heatmap cell is the Z value obtained by normalizing the relative abundance of species in each row (http://en.wikipedia.org/wiki/Standard_score), i.e., the Z value of a sample on a given classification is the difference between the relative abundance of the sample on that classification and the average relative abundance of all samples on that classification divided by the value obtained by dividing the standard deviation of the samples on that classification.

**FIG 3:**
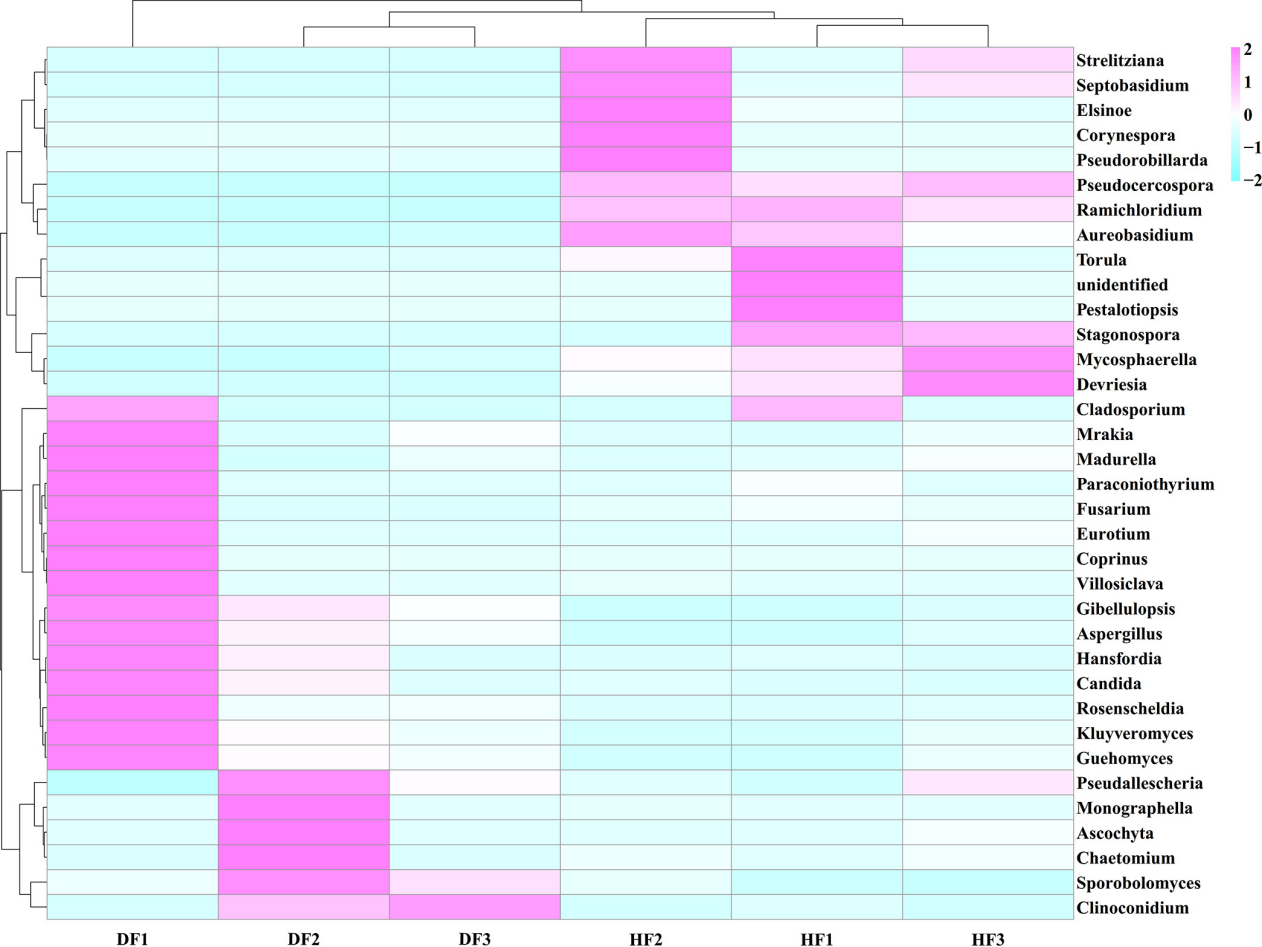
Heatmap of fungi in healthy and diseased fruits of *C. burmannii* at the genus level.

Based on the species annotation and abundance information of all samples at the order and genus levels, heatmap clustering analysis was performed on 35 orders or genera with higher abundance in six samples as a way to analyze the characteristics of the fungal community in diseased and healthy fruits (Fig. 2 and Fig. 3). The fungal community composition of the six samples was clustered into two large branches, and the three samples of diseased fruit and three samples of healthy fruit each had one branch. The fungal community compositions of the diseased and healthy fruit were significantly different. Moreover, there were significant differences between DF1 and the other samples in the diseased fruit group. Fungal communities in the DF and HF groups were significantly altered. At the order level (Fig. 2), in the DF group, Exobasidiales, Eurotiales, and Cystofilobasidiales were upregulated as the dominant groups, while the original dominant groups Capnodiales, Pleosporales, and Chaetothyriales were downregulated in the healthy fruit. At the genus level (Fig. 3), compared to the HF group, the dominant genera in the DF group, *Clinoconidium*, *Aspergillus*, and *Guehomyces*, were all upregulated, in addition to *Mrakia*, *Madurella*, *Paraconiothyrium*, *Fusarium*, *Eurotium*, *Coprinus*, *Villosiclava*, *Gibellulopsis*, *Hansfordia*, *Candida*, *Rosenscheldia*, *Kluyveromyces*, *Pseudallescheria*, *Monographella*, *Ascochyta*, *Chaetomium*, and *Sporobolomyces*, while the genera *Strelitziana*, *Septobasidium*, *Elsinoe*, *Corynespora*, *Pseudorobillarda*, *Pseudocercospora*, *Ramichloridium*, *Aureobasidium*, *Torula*, *Pestalotiopsis*, *Stagonospora*, *Mycosphaerella*, *Devriesia*, and *Cladosporium* exhibited pronounced downregulation. All of these results demonstrated a difference in the fungal community and composition between the healthy and diseased fruits.

### Biodiversity analysis of fungal communities in healthy and diseased fruits.

**FIG 4:**
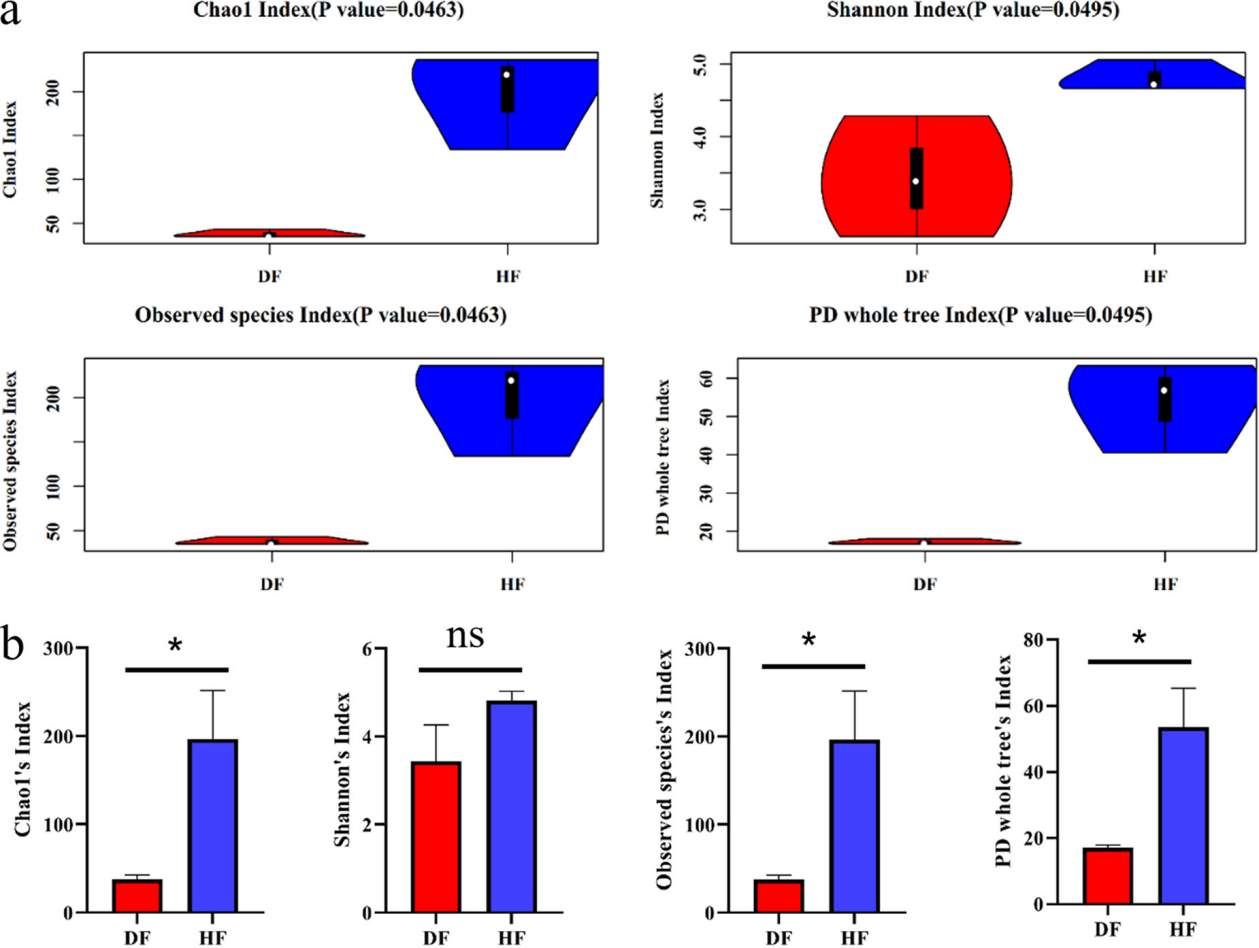
Alpha-diversity analysis of fungi from healthy and diseased fruit of *C. burmannii.* (a) Alpha diversity violin distribution between sample groups; the violin diagram in the figure is a combination of a boxplot and nuclear density diagram, the white dot is the median, the black box type range is from the lower quartile to the upper quartile, and the thin black line represents whiskers. The outer shape is the kernel density estimate. The *P* value at the top of the figure is the *P* value compared among the three groups, and it is generally believed that a *P* value less than 0.05 indicates significant differences between groups. (b) *, Significant differences by Student's *t* test (*, *P *< 0.05); ns, no significant difference.

**FIG 5:**
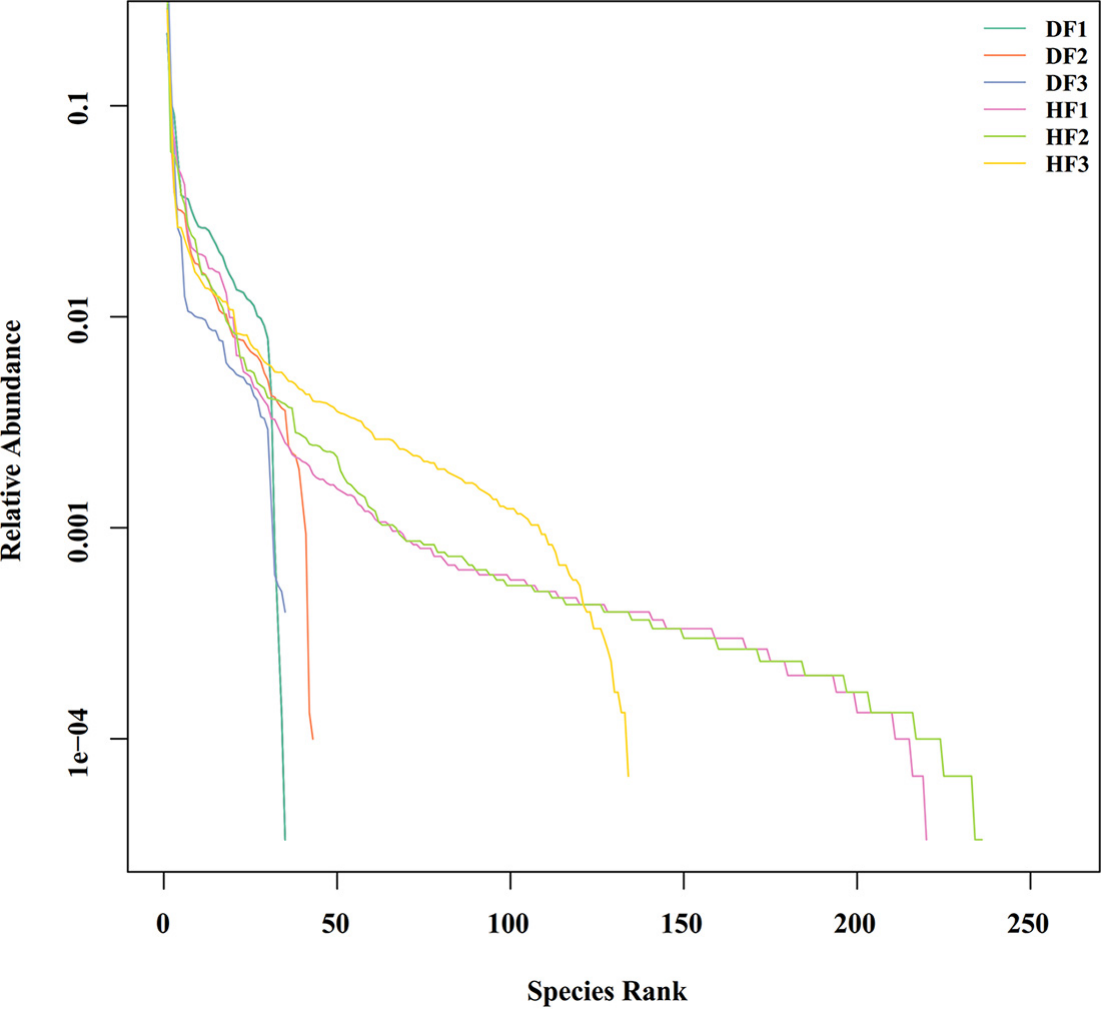
Rank abundance curve of different samples in *C. burmannii*. The abscissa is the serial number sorted by OTU abundance (rank), and the ordinate is the abundance of corresponding OTU (abundance). Different samples are represented by curves with different colors and linear lines.

The observed species, Chao1, Shannon, and (Phylogenetic diversity) PD whole-tree indices were used to determine the richness and diversity of the sampled species. Among them, the observed species index and Chao1 index measure the sample richness, while the Shannon and PD indices reflect the diversity of the sampled species. The higher these alpha diversity indices are, the more complex the diversity of the sample is. From the results of the alpha diversity violin plot (Fig. 4a) and Fig. 4b, it can be seen that the diversity of endophytic fungal communities in *C. burmannii* fruit in the HF group was significantly higher than that in the DF group (as observed for the Chao1, observed species, and PD whole-tree indices but not the Shannon index). The rank abundance curves (Fig. 5) showed that the abundance and evenness of the three samples in the HF group were stronger than those of samples in the DF group, with the species in HF2 and DF2 having the highest abundance and evenness of distribution in both the HF and DF groups. All of these observations may indicate that the species diversity of healthy fruit is more complex than that of diseased fruit.

The beta diversity indices can be used to evaluate fungal community differences between different samples. Bray-Curtis, weighted UniFrac, and unweighted UniFrac distances can be calculated from the OTU abundance information of samples. The beta diversity indices are indicators used to measure the dissimilarity coefficient between two samples, with smaller values indicating a smaller difference in species diversity between the two samples.

**FIG 6:**
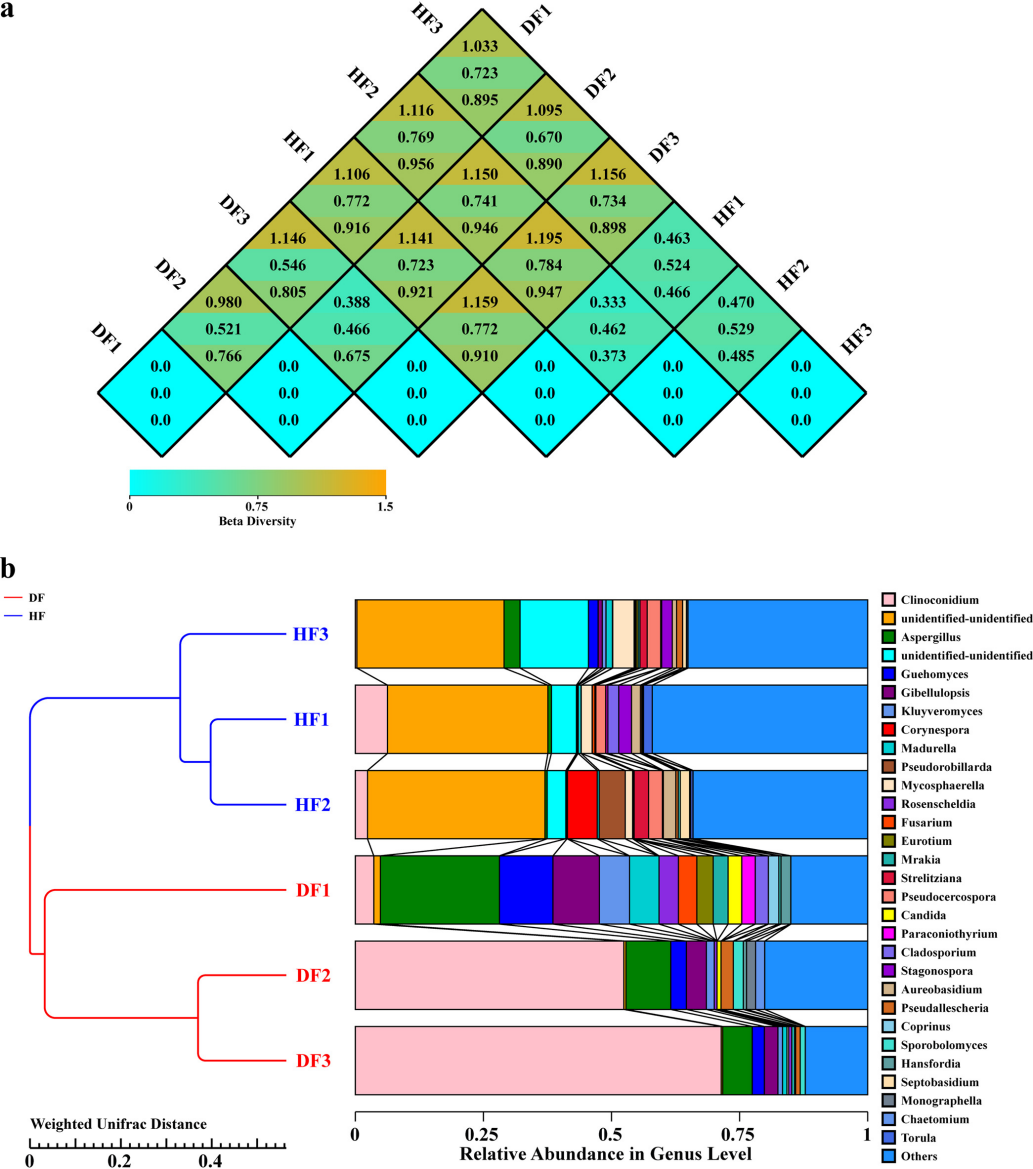
Beta-diversity analysis. (a) Heatmap of the beta diversity index. The numbers in the squares are the difference coefficients between the two samples. The smaller the difference coefficient is, the smaller the difference in species diversity. In the same grid, the top, middle, and bottom values represent the Bray-Curtis, weighted UniFrac and unweighted UniFrac distances, respectively. (b) UPGMA clustering analysis based on the weighted UniFrac distance on the left and relative abundance maps at the genus level on the right.

**FIG 7:**
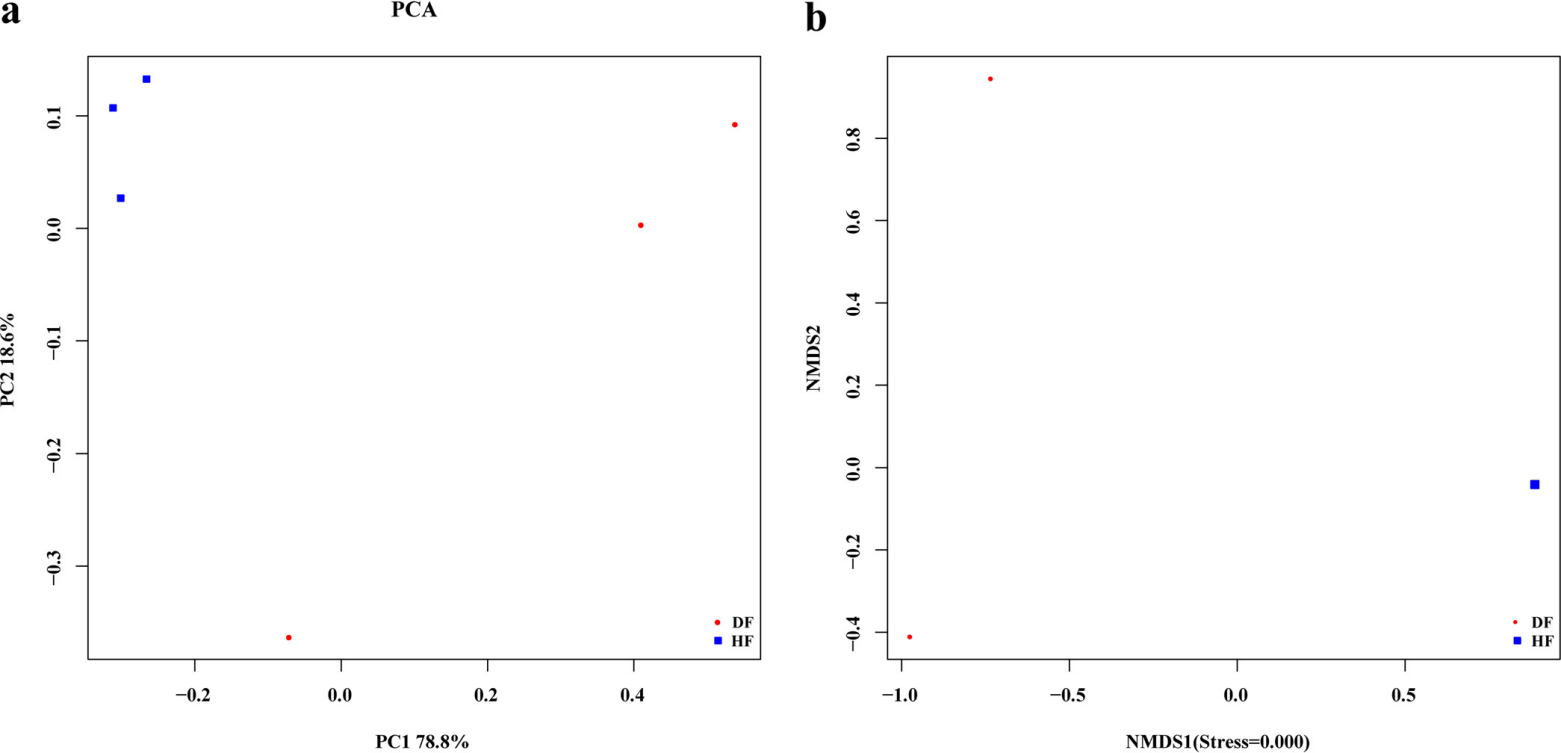
PCA and NMDS analysis of different samples in *C. burmannii*. (a) PCA results are presented based on the genus level. The abscissa (*x*) represents the first principal component, while the percentage represents the contribution of the first principal component to the sample difference. The ordinate (*y*) represents the second principal component, and the percentage represents the contribution of the second principal component to the sample difference. The Z coordinate represents the third principal component, and the percentage represents the contribution of the third principal component to the sample difference. Each dot in the figure represents a sample, and samples in the same group are indicated in the same color. (b) NMDS analysis diagram. Points in the figure represent samples, and the distance between points indicates the degree of difference. When stress is less than 0.2, NMDS analysis has a certain reliability.

Weighted UniFrac distances are associated with differences in species composition and numbers between samples. The mean values of the Bray-Curtis, weighted UniFrac, and unweighted UniFrac distances between different samples are shown in Fig. 6a. The mean distances in the HF group were 0.462 to 0.529 (weighted UniFrac), 0.333 to 0.470 (Bray-Curtis), and 0.373 to 0.485 (unweighted UniFrac). The mean distances in the DF group were 0.466 to 0.546 (weighted UniFrac), 0.388 to 1.146 (Bray-Curtis), and 0.675 to 0.805 (unweighted UniFrac). The mean distances between the HF and DF groups were 0.670 to 0.784 (weighted UniFrac), 1.033 to 1.159 (Bray-Curtis), and 0.890 to 0.956 (unweighted UniFrac). The average distance between the HF and DF groups was greater than the distance within the groups. An unweighted pair group method using average linkages (UPGMA) clustering tree (weighted UniFrac) of the species composition of the fungal groups in the healthy and diseased fruits of *C. burmannii* (Fig. 6b) was constructed, and the results were in general agreement with the principal-component analysis (PCA) results. At a genetic distance of 0.02, two branches were generated; the similarity between the species composition of healthy and diseased fruits was low, and the difference was significant. At a genetic distance of 0.2, there were two branches in diseased fruits as follows: DF2 and DF3 clustered into one category with high similarity and with no significant difference, and DF1 produced one branch with low similarity. The PCA results (Fig. 7a) showed that the first principal component (PC1) and the second principal component (PC2) explained 78.8% and 18.6% of the microflora of diseased fruit and healthy fruit from *C. burmannii*, respectively. The distance between the HF samples and DF samples was high, indicating that the community composition of healthy fruit was quite different from that of diseased fruit. The community compositions of the three samples in the HF group were similar, while the community compositions of the samples in the DF group were relatively different. As shown in Fig. 7b, the results of nonmetric multidimensional scaling analysis (NMDS) analysis showed good discrimination between the two groups, indicating that the fungal community composition of the HF group was different from that of the DF group. In addition, the composition of the fungal community also varied within the DF group.

### Identification and phylogenetic relationship analysis of culturable endophytic fungi.

**FIG 8:**
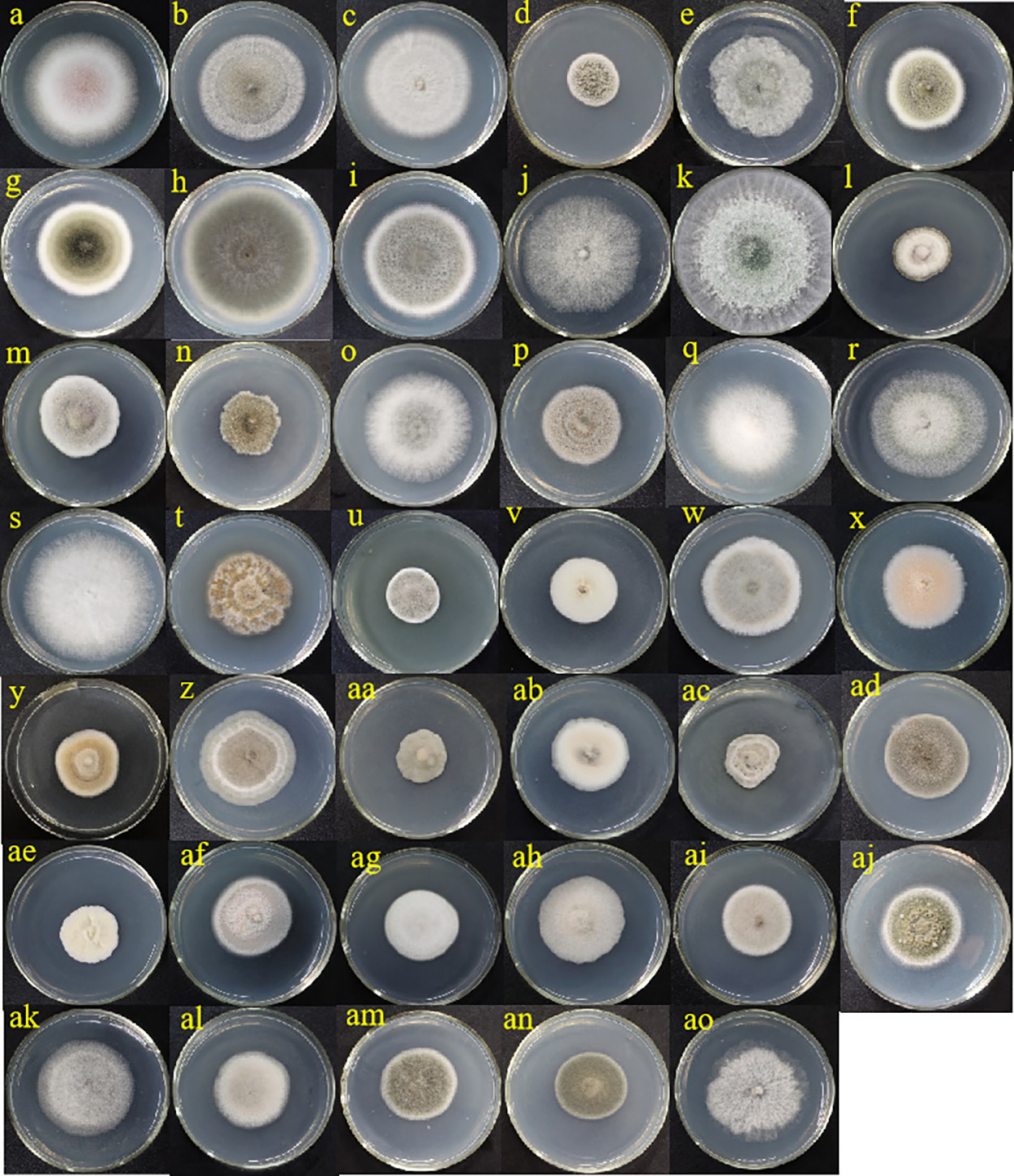
Endophytic fungi isolated from diseased and healthy fruit of *C. burmannii*. (a to ad) Endophytic fungal isolates Esf-1 to Esf-30, respectively. (ae to ao) Endophytic fungal isolates Cbf-1 to Cbf-11, respectively.

A total of 171 fungal isolates were isolated from healthy and diseased fruits of *C. burmannii*. Among them, 122 strains were isolated from diseased fruit, and 49 strains were isolated from healthy fruit. According to the molecular identification results, 41 strains were successfully identified as shown in Table 1 and Fig. 8, among which, 30 were from diseased fruit and 11 were from healthy fruit. All of the endophytic fungi were assigned to 23 genera as follows: *Acremonium*, *Allophoma*, *Alternaria*, *Aspergillus*, *Campylocarpon*, *Cladosporium*, *Colletotrichum*, *Curvularia*, *Diaporthe*, *Endomelanconiopsis*, *Fusarium*, *Medicopsis*, *Paraconiothyrium*, *Penicillium*, *Periconia*, *Phaeosphaeria*, *Phaeosphaeriopsis*, *Pseudocercospora*, *Pseudopithomyces*, *Setophoma*, *Teichospora*, *Trichoderma*, and *Xenoacremonium*. For endophytic fungi of diseased and healthy fruit, the dominant genus was *Colletotrichum* sp. Their partial ITS4-5.8S-ITS5 sequences were submitted to GenBank, with the accession numbers OK242732 to OK242772, and the closest related species with more than 97% similarity were obtained.

**TABLE 1:** Identification of endophytic fungi isolated from healthy and diseased fruits of *C. burmannii*

Strains	Accession no.	Macro- and microscopic identification	Similarity (%)	Closest related species accession no.
Cbf-1	OK242732	*Cladosporium* sp.	100.00	MF473111.1
Cbf-2	OK242733	*Acremonium* sp.	100.00	KT878351.1
Cbf-3	OK242734	*Colletotrichum* sp.	99.67	LC488852.1
Cbf-4	OK242735	*Periconia* sp.	99.65	MF435088.1
Cbf-5	OK242736	*Alternaria* sp.	100.00	MN955458.1
Cbf-6	OK242737	*Allophoma* sp.	100.00	MW036299.1
Cbf-7	OK242738	*Colletotrichum* sp.	100.00	KX197393.1
Cbf-8	OK242739	*Colletotrichum* sp.	100.00	MH930414.1
Cbf-9	OK242740	*Alternaria* sp.	100.00	MT453271.1
Cbf-10	OK242741	*Cladosporium* sp.	100.00	MF422154.1
Cbf-11	OK242742	*Diaporthe* sp.	100.00	MK942683.1
Esf-1	OK242743	*Fusarium* sp.	100.00	MK355724.1
Esf-2	OK242744	*Colletotrichum* sp.	100.00	MK211267.1
Esf-3	OK242745	*Fusarium* sp.	99.66	MN216215.1
Esf-4	OK242746	*Teichospora* sp.	97.89	MW764308.1
Esf-5	OK242747	*Endomelanconiopsis* sp.	100.00	GQ469968.1
Esf-6	OK242748	*Pseudopithomyces* sp.	100.00	MT186145.1
Esf-7	OK242749	*Alternaria* sp.	100.00	MN955458.1
Esf-8	OK242750	*Curvularia* sp.	100.00	JN116704.1
Esf-9	OK242751	*Colletotrichum* sp.	100.00	MG830360.1
Esf-10	OK242752	*Diaporthe* sp.	99.00	MH930433.1
Esf-11	OK242753	*Trichoderma* sp.	99.84	HQ608121.1
Esf-12	OK242754	*Setophoma* sp.	98.04	NR168153.1
Esf-13	OK242755	*Aspergillus* sp.	100.00	MN650839.1
Esf-14	OK242756	*Medicopsis* sp.	100.00	KM246269.1
Esf-15	OK242757	*Colletotrichum* sp.	100.00	KU642471.1
Esf-16	OK242758	*Periconia* sp.	99.50	KC954157.1
Esf-17	OK242759	*Fusarium* sp.	99.66	MT466521.1
Esf-18	OK242760	*Diaporthe* sp.	96.86	MH930427.1
Esf-19	OK242761	*Colletotrichum* sp.	100.00	AY266394.1
Esf-20	OK242762	*Campylocarpon* sp.	99.84	MK211263.1
Esf-21	OK242763	*Phaeosphaeria* sp.	98.47	MG827187.1
Esf-22	OK242764	*Phaeosphaeriopsis* sp.	99.46	GU017524.1
Esf-23	OK242765	*Alternaria* sp.	100.00	MK972909.1
Esf-24	OK242766	*Xenoacremonium* sp.	99.31	KM231833.1
Esf-25	OK242767	*Paraconiothyrium* sp.	99.32	EU715661.1
Esf-26	OK242768	*Pseudocercospora* sp.	99.82	KP896029.1
Esf-27	OK242769	*Phaeosphaeriopsis* sp.	99.07	KJ780762.1
Esf-28	OK242770	*Penicillium* sp.	100.00	MN788102.1
Esf-29	OK242771	*Phaeosphaeria* sp.	98.47	MG827187.1
Esf-30	OK242772	*Phaeosphaeriopsis* sp.	99.82	JQ936272.1

**FIG 9:**
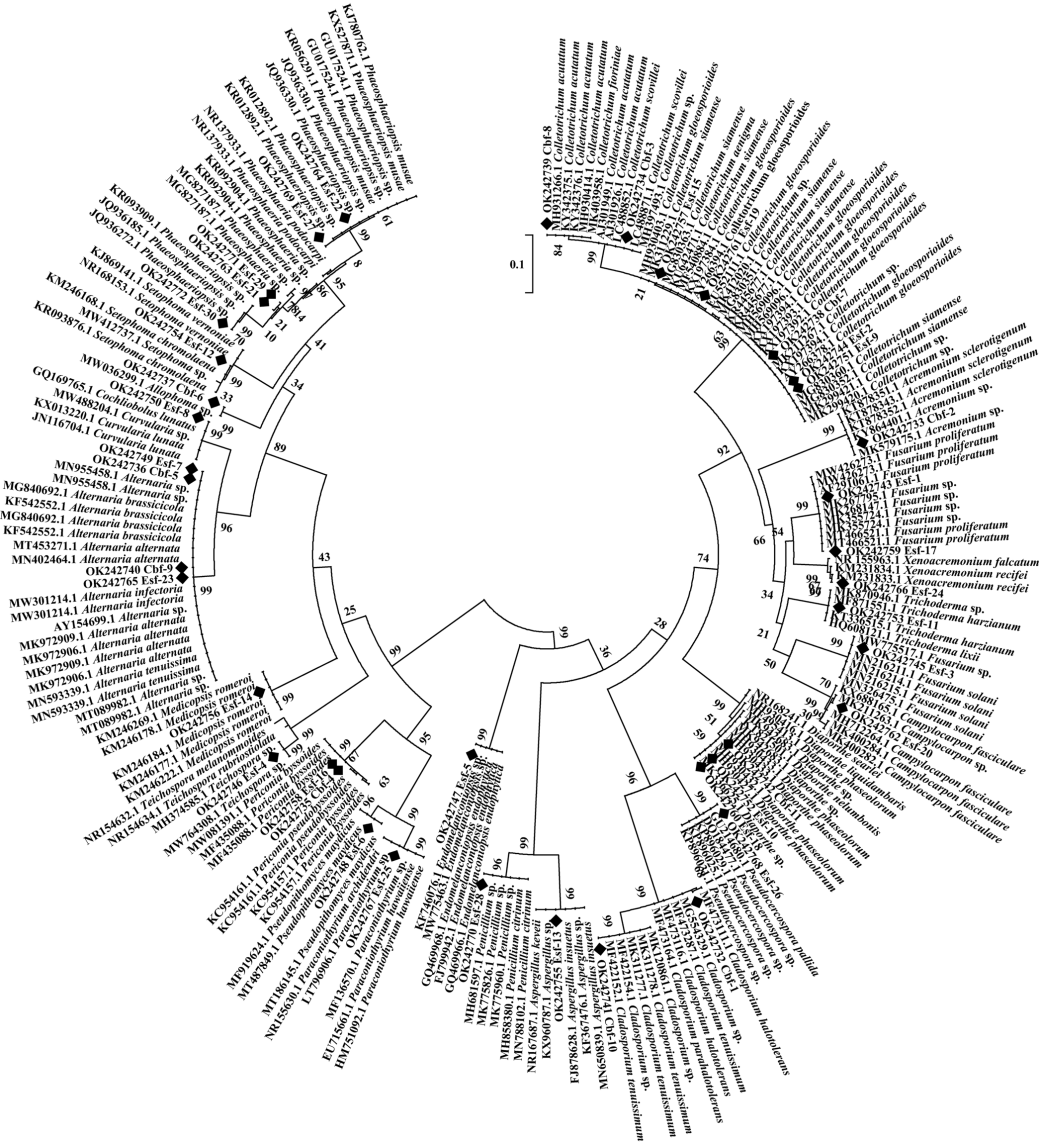
Phylogenetic tree for endophytic fungi of diseased and heathy fruit from *C. burmannii* based on the rDNA-ITS sequence.

All endophytic fungi were divided into 8 groups according to the phylogenetic tree results as follows: Botryosphaeriales, Cladosporiales, Diaporthales, Eurotiales, Glomerellales, Hypocreales, Mycosphaerellales, and Pleosporales. As shown in Fig. 9, the number of endophytic fungi in Pleosporales was the largest, followed by that in Glomerellales and Hypocreales. Diseased fruit may produce a greater variety of endophytic fungi than healthy fruit. Endophytic fungi from healthy fruit were classified into 5 groups, while endophytic fungi from diseased fruit were classified into 7 groups. Botryosphaeriales, Eurotiales, and Mycosphaerellales were unique to diseased fruit, while Cladosporiales was unique to healthy fruit. 41 endophytic fungi were divided into 23 different genera and 8 groups based on internal transcribed spacer (ITS) sequences, which reflected the diversity of endophytic fungi in diseased and healthy fruits of *C. burmannii*. In addition, the results also indicated the differences in endophytic fungi in diseased and healthy fruits.

### Antibacterial activity screening of the EtOAc extracts from the endophytic fungi.

The antibacterial activity of the crude extracts of endophytic fungi from 41 strains was determined by a thin-layer chromatography (TLC)-bioautography assay. The results of the antibacterial activity of 41 endophytic fungi are shown in Table S1 in the supplementary material, and the crude extracts of 41 isolates showed clear inhibitory activity against different tested bacteria. Specifically, the antibacterial activity of crude extracts of isolates in diseased fruit was stronger than that in healthy fruit. Among the endophytic fungi of diseased fruit of *C. burmannii*, all except Esf-2, Esf-4, Esf-10, Esf-12, Esf-25, Esf-26, Esf-27, and Esf-28 showed clear antibacterial activity against all of the tested bacteria, especially Esf-3, which showed strong antibacterial activity against all of the tested bacteria, and the diameter of the antibacterial plaque was more than 10 mm. Conversely, approximately half of the isolates from healthy fruit exhibited clear antibacterial activity against all of the tested bacteria, among which Cbf-6, Cbf-7, and Cbf-10 showed stronger antibacterial activity against all of the tested bacteria, and the diameter of the antibacterial plaque ranged from 5 mm to 10 mm. There was no significant difference in inhibitory activity against Gram-positive and Gram-negative bacteria. The range of the *R_f_* value could reflect the polarity of the active component in different crude extracts. It was also observed that the *R_f_* values with antibacterial activity in different crude extracts mostly ranged from 0.00 to 0.50. The results indicated that the substances with antibacterial activity in the crude extracts of isolates from diseased and healthy fruits were mainly compounds with low or moderate polarity.

### Correlation analysis of the microbiome, isolates, and antibacterial activity.

Based on fungal community and phylogenetic tree analysis, at the order level (Table 2), 9 upregulated and 16 downregulated endophytic fungi were isolated from diseased fruit (compared to the community from healthy fruit). Hypocreales was the order with the most upregulated endophytic fungi isolated (with 6 strains), while Pleosporales was the order with the greatest number of downregulated endophytic fungi isolated (with 14 strains). In combination with the antibacterial activity results, these findings showed that the endophytic fungi isolated from diseased fruit had strong antibacterial activity, exhibited by not only the upregulated genera, such as the well-known *Penicillium* and *Fusarium* (8), but also in the downregulated genera, suggesting that the downregulated genera may also contain active secondary metabolites. Even though Pleosporales were downregulated in diseased fruit, it was still the dominant flora in both healthy and diseased fruits, where strains of Pleosporales also showed clear antibacterial activity compared to the upregulated flora in diseased fruit, indicating that the downregulated flora of the fruit may contain strains with antibacterial activity or secondary metabolites involved in the response to pathogenic bacteria. Therefore, an endophytic fungal strain, Esf-14, of Pleosporales was selected to further demonstrate that it is feasible to search for active secondary metabolites in the downregulated flora of diseased fruit.

**TABLE 2:** Correlation analysis of the microbiome and isolates

Order	HF (accession no.)	DF (accession no.)	Up/down
Hypocreales	Cbf-2 *Acremonium* sp. (OK242733)	Esf-1 *Fusarium* sp. (OK242743)	Up
		Esf-3 *Fusarium* sp. (OK242745)	
		Esf-11 *Trichoderma* sp. (OK242753)	
		Esf-17 *Fusarium* sp. (OK242759)	
		Esf-20 *Campylocarpon* sp. (OK242762)	
		Esf-24 *Xenoacremonium* sp. (OK242766)	
Botryosphaeriales	NA^*a*^	Esf-5 *Endomelanconiopsis* sp. (OK242747)	Up
Eurotiales	NA	Esf-13 *Aspergillus* sp. (OK242755)	Up
		Esf-28 *Penicillium* sp. (OK242770)	
Pleosporales	Cbf-4 *Periconia* sp. (OK242735)	Esf-4 *Teichospora* sp. (OK242746)	Down
	Cbf-5 *Alternaria* sp. (OK242736)	Esf-6 *Pseudopithomyces* sp. (OK242748)	
	Cbf-6 *Allophoma* sp. (OK242737)	Esf-7 *Alternaria* sp. (OK242749)	
	Cbf-9 *Alternaria* sp. (OK242740)	Esf-8 *Curvularia* sp. (OK242750)	
		Esf-12 *Setophoma* sp. (OK242754)	
		Esf-14 *Medicopsis* sp. (OK242756)	
		Esf-16 *Periconia* sp. (OK242758)	
		Esf-21 *Phaeosphaeria* sp. (OK242763)	
		Esf-22 *Phaeosphaeriopsis* sp. (OK242764)	
		Esf-23 *Alternaria* sp. (OK242765)	
		Esf-25 *Paraconiothyrium* sp. (OK242767)	
		Esf-27 *Phaeosphaeriopsis* sp. (OK242769)	
		Esf-29 *Phaeosphaeria* sp. (OK242771)	
		Esf-30 *Phaeosphaeriopsis* sp. (OK242772)	
Diaporthales	Cbf-11 *Diaporthe* sp. (OK242742)	Esf-10 *Diaporthe* sp. (OK242752)	Down
		Esf-18 *Diaporthe* sp. (OK242760)	
Cladosporiales	Cbf-1 *Cladosporium* sp. (OK242732)	NA	NA
	Cbf-10 *Cladosporium* sp. (OK242741)		
Glomerellales	Cbf-3 *Colletotrichum* sp. (OK242734)	Esf-2 *Colletotrichum* sp. (OK242744)	Down
	Cbf-7 *Colletotrichum* sp. (OK242738)	Esf-9 *Colletotrichum* sp. (OK242751)	
	Cbf-8 *Colletotrichum* sp. (OK242739)	Esf-15 *Colletotrichum* sp. (OK242757)	
		Esf-19 *Colletotrichum* sp. (OK242761)	
Mycosphaerellales	NA	Esf-26 *Pseudocercospora* sp. (OK242768)	Down

a NA, data are not available.

### Species identification results for the endophytic fungus Esf-14.

**FIG 10:**
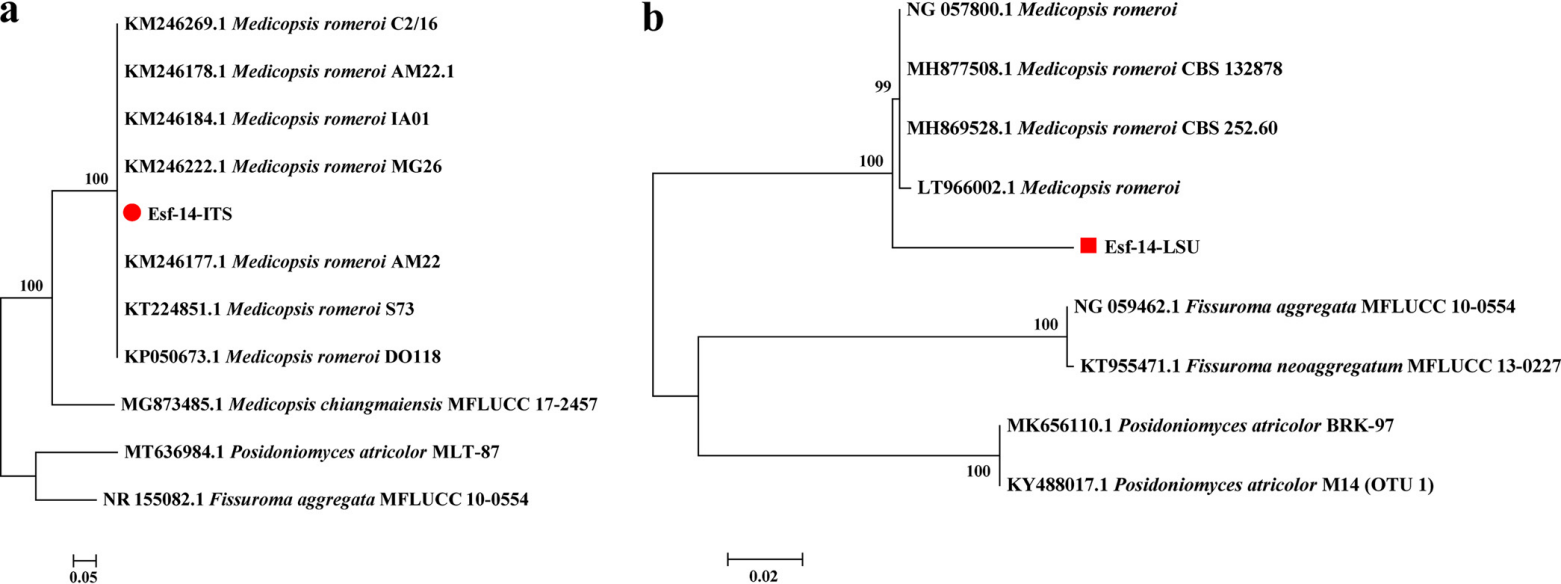
Construction of a phylogenetic tree of strain Esf-14 and other similar species based on ITS or LSU gene sequences. (a) Single gene phylogenetic tree (ITS). (b) Single gene phylogenetic tree (LSU).

Phylogenetic trees based on ITS or (Large subunit ribosomal RNA gene sequence) LSU were constructed using strains of *Medicopsis* sp. and other similar strains as outgroups (Fig. 10). The results for strain Esf-14 were consistent among these phylogenetic tree analyses. The ITS-based single gene phylogenetic tree indicated that strain Esf-14 was located in the same evolutionary branch as *Medicopsis romeroi* with a spreading value of 100. The LSU-based single gene phylogenetic tree also showed that Esf-14 was closely related to *M. romeroi*. The results of the multigene association tree suggested that both Esf-14 and *M. romeroi* were on the same evolutionary branch, with spreading values of 100 (ITS) and 92 (LSU), respectively. Consequently, strain Esf-14 could be identified as *M. romeroi*.

### Purification and structure elucidation.

**FIG 11:**
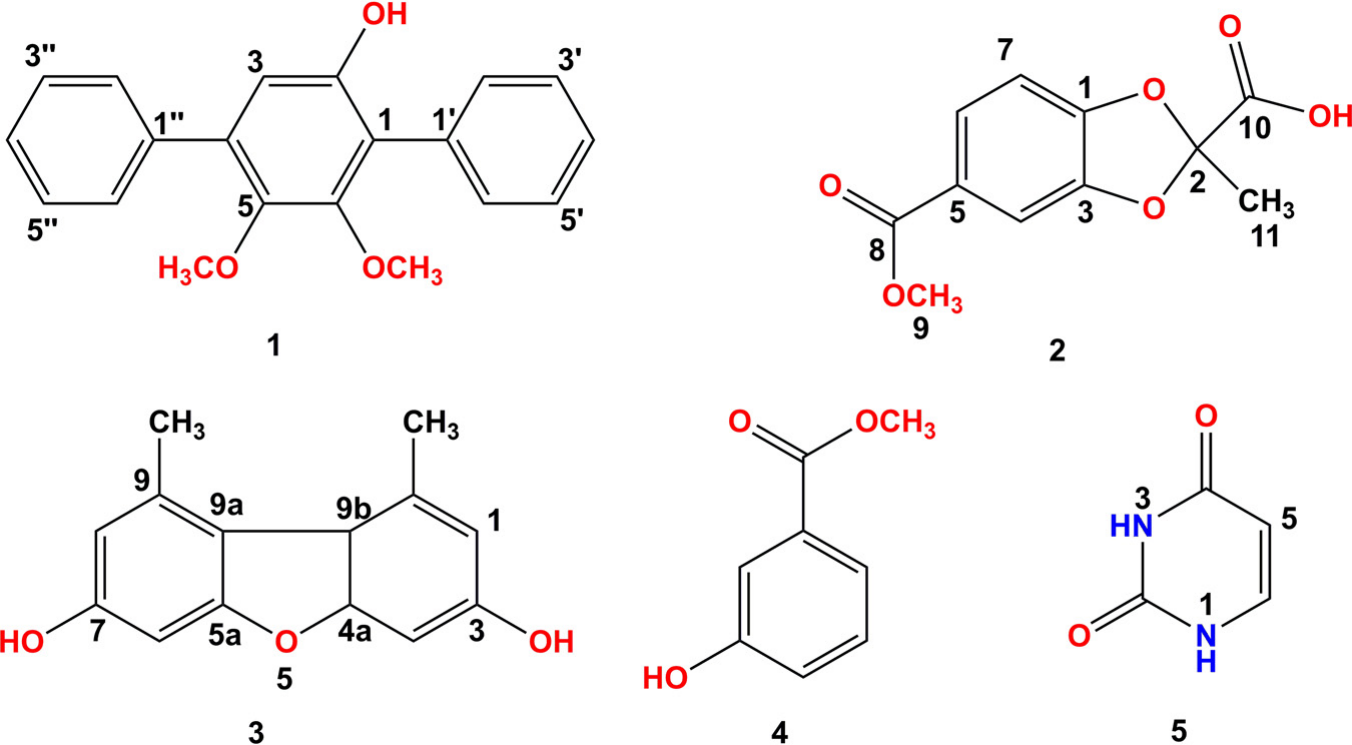
Chemical structures of isolated compounds 1 to 5.

Five compounds 1 to 5 were further separated and purified from the ethyl acetate extract of stain Esf-14 using chromatographic techniques. Of these, compounds 3 to 5 are known compounds, while 1 and 2 are probably new compounds. Then, structural characterization of these compounds was performed (Fig. 11). The chemical structures of compounds 1 to 5 were elucidated by high-resolution electrospray ionization mass spectrometry (HR-ESI-MS) and one-dimensional (1D) and two-dimensional (2D) nuclear magnetic resonance (NMR) experiments (correlation spectroscopy [COSY], heteronuclear single quantum coherence [HSQC], and heteronuclear multiple-bond correlation [HMBC]).

5,6-Dimethoxy-[1′,1:4,1″-terphenyl]-2-ol (compound 1) was isolated as a colorless waxy solid. It showed a prominent pseudomolecular peak at *m/z* 307.1331 [M+H]^+^ (calculated for C_20_H_19_O_3_, 307.1329) and 329.1132 [M+Na]^+^ (calculated for C_20_H_18_O_3_Na, 329.1148) in the positive HR-ESI-MS (see Fig. S5 in the supplemental material), indicating its molecular formula as C_20_H_18_O_3_ with 12 degrees of unsaturation. The UV spectrum (see Fig. S6 in the supplemental material) showed absorption peaks at 200, 247, and 256 nm, which were similar to those of 4,4″-deoxyterphenyllin (27). The ^1^H NMR, ^13^C NMR (Table 3), and HSQC spectra suggested the compound consists of two monosubstituted benzene rings and one pentasubstituted benzene ring. For the ^1^H NMR spectrum, the absorption for the aromatic protons of pentasubstituted benzene appears as a strong singlet at 6.78 ppm. Three O-substituted aromatic quaternary carbon (δ_C_ 157.30, 154.40, 153.02, and 145.87), 10 methine carbon and two methoxy carbon signals (δ_C_ 61.04, 60.84 and 56.43) were shown in ^13^C NMR. The HMBC signals of H-3/C-2 and C-5, and the nuclear Overhauser effect spectroscopy (NOESY) signal of 5-OCH_3_/6-OCH_3_ indicated that the structure of compound 1 was 5,6-dimethoxy-[1′,1:4,1″-terphenyl]-2-ol.

**TABLE 3:** ^1^H (600 MHz) and ^13^C (150 MHz) NMR data of compound 1 (CDCl_3_, *J* in Hz)

Position	δ_C_, type	δ_H_	HMBC
1	149.13		
2	122.45		
3	151.44		
4	144.56		
5	136.41		
6	112.07	6.78 (s, 1H)	C-2, C-5, C-1″
1′	132.62		
2′/6′ 3″/5″	130.58, 128.39	7.44 (m, 4H)	C-1, C-4′
3′/5′	129.46	7.53 (m, 2H)	C-1′
4′	128.50	7.44 (m, 1H)	C-2′, C-6′
1″	138.10		
2″/6″	129.34	7.59 (m, 2H)	C-4, C-4″
4″	127.53	7.36 (m, 1H)	C-2″, C-6″
5-OCH_3_	61.17	3.68 (s, 3H)	C-5
6-OCH_3_	61.04	3.59 (s, 3H)	C-6

**FIG 12:**
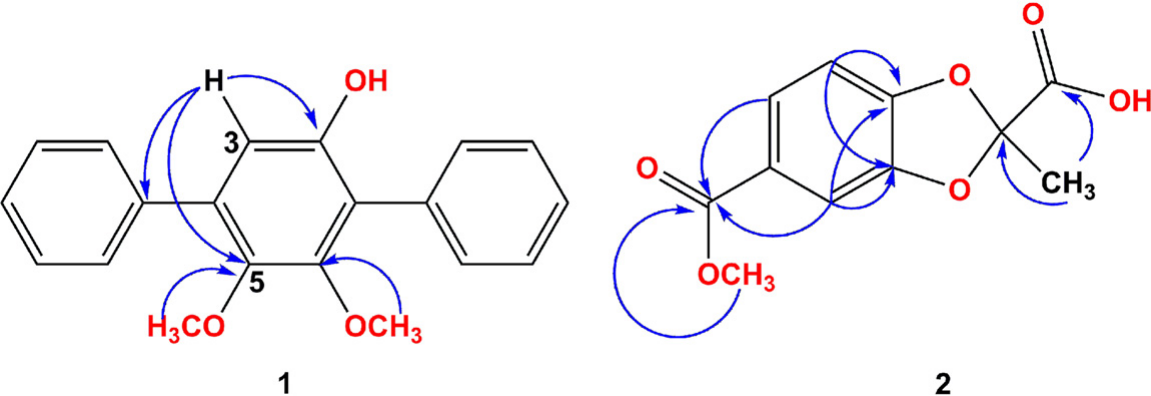
Key HMBC (H-C) correlations of compounds 1 and 2.

5-(Methoxycarbonyl)-2-methylbenzo[d][1,3]dioxole-2-carboxylic acid (compound 2) was isolated as a colorless waxy solid. Its molecular formula was established as C_11_H_10_O_6_ (seven degrees of unsaturation) based on the prominent pseudomolecular ion peak at *m/z* 237.0406 [M-H]- in the HR-ESI-MS spectrum (see Fig. S12 in the supplemental material). The ^1^H NMR spectrum (Table 4) of 2 showed the presence of one ABX trisubstituted benzene at δ_H_ 7.55 (dd, *J *= 1.8, 8.4 Hz, 1H, H-6), 7.34 (d, *J *= 1.2 Hz, 1H, H-4), and 6.99 (d, *J *= 7.8 Hz, 1H, H-7). The ^13^C NMR (Table 4) and HSQC spectra suggested 11 carbon signals, including two carbonyl carbons (δ_C_ 167.36, 165.56), six aromatic carbons (δ_C_ 151.30, 147.49, 124.83, 123.28,108.25, 107.97), three methines, one methoxy carbon (δ_C_ 52.01), and one methyl carbon (δ_C_ 21.85). Except for one benzene ring and two carbonyl groups, one degree of unsaturation was unassigned, which indicated that there was a ring. Thus, the remaining ^13^C NMR signal (δ_C_ 113.57) was a assigned to be quaternary carbon connecting with two oxygen atoms, which formed a five-membered ring. The HMBC correlations (Fig. 12) of H-11/C-2 and C-10, H-9/C-8 suggested that compound 2 has the structure shown in Fig. 11.

**TABLE 4:** ^1^H (600 MHz) and ^13^C (150 MHz) NMR data of compound 2 (DMSO-*d_6_*, *J* in Hz)

Position	δ_C_, type	δ_H_	HMBC
1	151.30, C		
2	113.57, C		
3	147.49, C		
4	108.25, CH	7.34 (d, *J *= 1.2 Hz, 1H)	C-6, C-3, C-1, C-8
5	123.28, C		
6	124.83, CH	7.55 (dd, *J *= 1.8, 8.4 Hz, 1H)	C-7, C-4, C-1, C-8
7	107.97, CH	6.99 (d, *J *= 7.8 Hz, 1H)	C-5, C-3, C-1
8	165.56, C		
9	52.01, CH_3_	3.80 (s, 3H)	C-8
10	167.36, C		
11	21.85, CH_3_	1.80 (s, 3H)	C-10, C-2

Known compounds 3 to 5 were identified according to their spectroscopic data (^1^H NMR, ^13^C NMR, and 2D NMR) as well as comparison with literature data. The clear UV and NMR spectra of known compounds are also provided in the Supplementary Materials to further confirm the structure (see Fig. S19 to S28 in the supplementary material).

## DISCUSSION

The community composition of plant endophytic fungi is important for the biodiversity of natural ecosystems (28). These endophytic fungi not only enhance plant growth performance (29) and improve plant tolerance to biotic and abiotic factors in the environment (27, 30) but also are involved in waste decomposition and nutrient cycling (31). However, when infected with pathogenic bacteria, the composition of the fungal community in plant tissues changes, which in turn causes changes in various metabolic functions (32). Therefore, it is necessary to understand the composition and structural characteristics of fungal communities in healthy and diseased fruits of *C. burmannii*.

Previous studies have suggested that endophytic fungi are affected by environmental factors, tissue type, and plant age (33). In addition, variations in the communities on leaves have been reported (14), while this study reported for the first time the variation in endophytic fungal communities between healthy and diseased fruits of *C. burmannii* using high-throughput sequencing analysis. The structure and composition of fungal communities in healthy and diseased fruits of *C. burmannii* were significantly different at the phylum, class, order, or genus level. Fungal species richness was significantly lower in diseased fruit compared to healthy fruit, which indicated a decrease in the number of fungi in the communities, and some differences in fungal community diversity were observed in the diseased fruit group. This difference may be related to the different durations of pathogenic bacterial infection (34).

The main microbes in the diseased fruit, namely, *Clinoconidium*, *Cladosporium*, *Mrakia*, *Madurella*, *Paraconiothyrium*, *Fusarium*, *Eurotium*, *Coprinus*, *Villosiclava*, *Gibellulopsis*, *Aspergillus*, *Hansfordia*, *Candida*, *Rosenscheldia*, *Kluyveromyces*, *Guehomyces*, *Pseudallescheria*, *Monographella*, *Ascochyta*, *Chaetomium*, and *Sporobolomyces*, exhibited significant upregulation, and the pathogenic group *Clinoconidium* had the highest relative abundance, which likewise suggested that *Clinoconidium* may be the main pathogen of *C. burmannii* fruit (35). The relative abundance of *Clinoconidium* in the microbiota may be related to the regulatory function of plant fungal communities or pathogenesis. In this study, the genus *Clinoconidium*, which includes the causative agent of *C. burmannii* powdery mildew, was found to be present in both healthy and diseased fruits (35), and both were dominant, with the only difference being that the abundance of *Clinoconidium* in healthy fruit was significantly lower than that in diseased fruit. *Clinoconidium* did not show symptoms of disease, although it was present in healthy fruit, which may be related to the higher abundance of the fungal community in healthy fruit, as it has been shown that the diversity of fungal communities might play an important role in reducing invasion by pathogens (28).

Recent studies have indicated that volatile chemicals in plants are regulated by the microbiota (36). We previously reported dramatic differences in the volatile composition of healthy and diseased fruits of *C. burmannii* (26). The major volatile components in healthy fruit were α-caryophyllene (32.94%) and *L*-caryophyllene (17.75%). After fruit infection, the major volatiles in the diseased fruit were β-guaiene (10.01), (−)-β-cadinene (8.64%), *L*-caryophyllene (5.78%), pinene (5.68%), and α-caryophyllene (5.25%). The differences in volatile chemical composition might be related to the turnover of the fungal community. Infection of healthy fruit by pathogenic bacteria was originally thought to be inhibited by toxic substances produced by the plant, resulting in changes in the chemical composition of the fruit (37–39); however, the results of the present study could also explain why differences in chemical composition in plants may also be associated with fungal community turnover. All of these results illustrated that the replacement process of fungal communities of diseased and healthy fruit might generate more abundant bioactive resources.

Numerous studies have shown that when plants are stressed by biotic factors such as pathogens, the fungal community in the tissues changes in response to the stimulus (40, 41), while the plant endophytic fungi also secrete a greater variety of bioactive substances to counteract the adverse factors. Fungal groups play a more important role in the plant disease coexistence network than in the healthy network (25) as also demonstrated in our study. The present study also supported the above results by measuring the antibacterial activity of 41 endophytic fungal secondary metabolites and further found that the fungal populations secreting active secondary metabolites were distributed in both healthy and diseased fruits. In addition, the endophytic fungal secondary metabolites in diseased fruit exhibited stronger antibacterial activity than those in healthy fruit. Moreover, interestingly, as in previous studies, researchers have focused more on healthy plant tissues and less on the bioactive function of fungal secondary metabolites in diseased tissues (24). All endophytic fungal strains were classified by phylogenetic tree analysis into 8 orders and 23 genera, which responded to the diversity and exploitability of culturable endophytic fungi in fruits. The correlation analysis of fungal communities, phylogenetic trees, and antibacterial activity revealed that fungi with antibacterial activity in diseased fruit were distributed in both upregulated or downregulated groups, an unexpected finding that suggested the existence of an interesting pathway via which not only upregulated but also downregulated stress-related microbes in diseased fruit secrete active secondary metabolites. The discovery may be useful for understanding the function of the microbiota and the study of active secondary metabolites.

Pleosporales is the largest and most diverse order in Dothideomycetes, which includes a variety of endophytic fungi, and the strains in this group produce abundant secondary metabolites that are often used to develop new antimicrobials and chemical pesticides or derive biopesticide molecules (42). Subsequently, the active strain Esf-14, identified as *M. romeroi* in Pleosporales, was screened by phylogenetic tree, fungal community, and antibacterial activity analyses, and two new and three known compounds were isolated from this strain. The structures of the new compounds were elucidated for the first time. One of the new compounds 1 (5,6-dimethoxy-[1′,1:4,1″-terphenyl]-2-ol) is a new *o*-terphenyl analog, which is an aromatic compound consisting of three benzene rings with three isomers, namely, *o*-terphenyl, *m*-terphenyl, and *p*-terphenyl, with *p*-terphenyl and m-terphenyl predominating in nature. These compounds often possess good biological activity and play an important role in pharmaceutical and industrial applications (43, 44). The newly discovered compound in this study is of great research value as a triplet biphenyl compound and is perhaps equally rich in biological activity.

In this study, we reported for the first time the diversity of fungal communities on diseased and healthy fruit of *C. burmannii* and antibacterial activity of secondary metabolites from artificially cultivatable fungi. The structure and composition of fungal communities on diseased fruit were altered compared to those on healthy fruit. Although the fungal community diversity on diseased fruit was lower than that on healthy fruit, the secondary metabolites produced by the fungi in these fruits appeared to have stronger antibacterial functions. Five compounds were obtained for the first time from the active strain *M. romeroi* (Esf-14; GenBank accession number OK242756) isolated from *C. burmannii* diseased fruits, including two new compounds (5,6-dimethoxy-[1′,1:4,1″-terphenyl]-2-ol [compound 1] and 5-(methoxycarbonyl)-2-methylbenzo[d][1,3]dioxole-2-carboxylic acid [compound 2]) and three known compounds. All of these results are essential for understanding the active functions of the microbiota and developing novel bioactive resources.

## MATERIALS AND METHODS

### Plant material.

The plant material for this study was obtained from Huolu Mountain (23°19' N, 113°39' E, 600 ha, 321.8 m above sea level) in Guangzhou, Guangdong Province, southern China, which is located in a subtropical monsoon climate zone with an average annual temperature of 24°C and an average annual humidity of 72% to 90%. Healthy and diseased *C. burmannii* fruits were collected from Huolu Mountain in 2016 (see Fig. S1 in the supplemental material). The fruits (three biological replicates each, for a total of six samples) were immediately placed in well-labeled sterile plastic bags, transported back to the laboratory in ice boxes, and then stored at −80°C until being used for DNA extraction. The material for isolation of endophytic fungi was also collected as described above, but the strains were isolated immediately after shipment back to the laboratory.

### DNA extraction, amplification, and sequencing.

The preserved plant material was removed from the −80°C freezer, thawed at room temperature, rinsed with tap water for 15 min, transferred to a sterile bench, treated with 70% anhydrous ethanol for 5 min, soaked in 2.5% NaClO solution (0.1% Tween 80) for 5 min, treated with 70% anhydrous ethanol for 3 min, and finally washed three times with sterile water for 5 min each time. After drying on sterile filter paper, DNA was extracted from the fruit using the cetyltrimethylammonium bromide (CTAB) method (28). PCR amplification of the ITS1-ITS2 regions of the obtained DNA was performed using the fungal universal primers ITS1F (CTTGGTCATTTAGAGGAAGTAA) and ITS2R (GCTGCGTTCTTCATCGATGC). The PCR amplification conditions were as follows: 35 cycles of predenaturation at 94°C for 30 s, annealing at 52°C for 45 s, and extension at 72°C for 1 min; extension at 72°C for 10 min; and then storage at 4°C. The amplified products were detected by 2% agarose gel electrophoresis, and the qualified PCR products were further purified and quantified to obtain the sequencing library, which was sequenced by Shenzhen HENGCHUANG Gene Technology Co., Ltd. (China).

### Bioinformatics analysis.

Raw data obtained by using the Illumina MiSeq/HiSeq sequencing platform were preprocessed to filter out low-quality data. First, the raw data were split using a Perl script, and then the reads of each sample were spliced using FLASH (v1.2.7) (http://ccb.jhu.edu/software/FLASH/) to obtain the raw tag data. The raw data were processed using Quantitative Insights into Microbial Ecology (QIIME V1.9.0; http://qiime.org/index.html) to truncate raw tags from the first low-quality site where the number of consecutive low-quality (default quality threshold, ≤3) bases reached a set length (default length value of 3). Tags in which the length of consecutive high-quality bases was less than 75% of the total tag length were further filtered out. The clean tag sequences obtained after these processes were compared against the Gold database (http://drive5.com/uchime/uchime_download.html) with the UCHIME algorithm (http://www.drive5.com/usearch/manual/uchime_algo.html) to detect chimeric sequences, and after removing the chimeras, effective tags were finally obtained. The valid sequences of all of the samples were computed using UPARSE software (UPARSE v7.0.1001; http://drive5.com/uparse/) to classify operational taxonomic units (OTUs) at a 97% threshold. Representative OTUs were classified by QIIME software combined with the RDP classifier method. The representative OTUs were compared with the Greengenes, RDP, Silva, and Unite databases to obtain species annotation information (the confidence threshold was 0.8 or higher by default). The proportion of annotations and the relative abundance of species at each taxonomic level of the OTUs were determined using R software. The effective sequences of all of the samples were computed using UPARSE software (UPARSE v7.0.1001; http://drive5.com/uparse/) at a 97% threshold to classify OTUs. Representative OTUs were compared by QIIME software combined with the RDP classifier method. The representative OTUs were compared with the Greengenes, RDP, Silva, and Unite databases to obtain species annotation information (the confidence threshold was 0.8 or higher by default). The proportion of annotations and the relative abundance of species at each taxonomic level of the OTUs were determined using R software. Phylogenetic analysis of OTUs was performed using KRONA software, QIIME software, and Perl scripts. A heatmap of relative abundance at the genus level using R software was generated, and intersample and interspecies clustering analysis was performed. QIIME software was used to calculate four diversity indices (observed species, Chao, Shannon, and PD whole tree) and their rarefaction curve, among which the PD whole-tree index needed OTU phylogenetic tree data. Rank abundance was also established with QIIME software at the same time. Three kinds of beta diversity indices (Bray Curtis, weighted UniFrac, unweighted UniFrac) were calculated by QIIME software, and heatmapping of the beta diversity indices was completed by using native Perl and SVG software. Principal-component analysis (PCA) and nonmetric multidimensional scaling analysis (NMDS) were performed with R software. A cluster tree of the samples was established by using QIIME software with the UPGMA method. Differences in alpha diversity indices between healthy and diseased fruits were determined using Student's *t* test.

### Isolation and culture of the endophytic fungi.

The isolation of endophytic fungi from healthy and diseased fruits of *C. burmannii* was carried out according to the method used by Wang et al. (45) with some modifications. The healthy and diseased fruits of *C. burmannii* were washed with running water, treated with 70% ethanol for 30 s and 0.2% mercury chloride for 20 min, washed with sterile water for 5 min, and dried on sterile filter paper. The fruits were cut into pieces of approximately 0.3 cm by 0.3 cm by 0.3 cm and placed on peptone-dextrose agar (PDA) medium (with 500 μg/mL streptomycin sulfate). Three pieces were placed in one dish and incubated in a constant-temperature incubator at 28°C for 30 days. A few mycelia were selected from the edge of the colony in the petri dish, in which the mycelia grew vigorously, and then inoculated on PDA medium. Repeated purification was carried out until the colony morphology was stable and consistent with no major changes. The purified strains were stored at 4°C until further use.

### Molecular biological identification of isolates.

DNA from the isolates was extracted according to the protocol for the fungal genomic DNA extraction kit. ITS-ribosomal DNA (rDNA) was amplified by PCR, and the primers used for sequencing were the universal primers ITS4 (5′-TCCTCCGCTTATTGATATGC-3′) and ITS5 (5′-GGAAGTAAAAGTCGTAACAAGG-3′). The PCR system was as follows: 1 μL of ITS4, 1 μL of ITS5, 3 μL of DNA extract, 25 μL of PCR master mix, and 20 μL of sterile deionized water. The PCR amplification conditions were as follows: denaturation at 94°C for 3 min, followed by 30 cycles of denaturation at 94°C for 30 s, annealing at 52°C for 30 s, and extension at 72°C for 1 min, with a final extension at 72°C for 8 min and storage at 4°C. Then, the PCR products were sequenced and purified by Shanghai Biological Engineering Co., China. DNAMAN software was used to determine the cDNA sequences, and forward and reverse primers sequences were added to both ends of the complementary sequences to obtain the complete sequence. To obtain the accession numbers, the ITS-rDNA sequences were submitted to the GenBank database (National Center for Biotechnology Information; http://www.ncbi.nlm.nih.gov) and analyzed by the BLAST program. After MAFTT (version 7.0) processing, all of the sequences were analyzed by MEGA7 software with the maximum likelihood method.

### Fungal material and preparation of EtOAc extracts.

Different crude extracts of isolates were prepared according to a previous report (46). First, the selected isolates were inoculated into triangular bottles (50 mL) containing 20 mL of potato dextrose broth (PDB) medium, with each fungus culture grown in two bottles. Then, all of the cultures were added to sterilized rice culture medium at 25°C and grown for 60 days. Second, each fermentation product was extracted three times in methanol for 7 days each time. Finally, the fermentation liquid was filtered and dried under vacuum with reduced pressure to obtain the ethyl acetate extract. Next, the crude extracts were dissolved in water, extracted with an equal volume of ethyl acetate 3 to 5 times, and then decompressed and concentrated again to obtain a crude ethyl acetate extract, which was collected in a clean bottle and stored at 4 to 5°C until further use.

### Antibacterial activity of crude EtOAc extract.

The antibacterial activity of the ethyl acetate extracts of 30 endophytic fungi from diseased fruit and 11 endophytic fungi from healthy fruit was determined using the Thin layer chromatography (TLC)-bioautography method described in our previous report. The formula *R_f_* = *D*_1_/*D*_2_ was used to characterize the polarity of the active substance, and the antibacterial activity was evaluated by the size of the inhibitory spot diameter (45).

### Multigene identification in strain Esf-14.

The isolate Esf-14 was identified as *Medicopsis* sp. according to the ITS-based phylogenetic tree analysis of the 41 strains, and further species identification of the strain was performed using ITS and LSU (LROR, 5′-ACCCGCTGAACTTAAGC-3′ and LR5, 5′-TCCTGAGGGAAACTTCG-3′) genes. The sequences of ITS and LSU (*Medicopsis* sp.) were blasted separately against the NCBI nucleotide database and downloaded, and the sequences of other species of the same genus or similar species were used as reference sequences. The combinatorial matrix-based comparison was then performed using MAFFT version 7 (https://mafft.cbrc.jp/alignment/software/algorithms/algorithms.html) after merging the results with the sequence data. The best tree building model was identified using MEGA 7.0 (GTR+G+I), and the bootstrap method was used with 1,000 bootstrap replicates (47).

### Fermentation and extraction of the endophytic fungi Esf-14.

The crude extract of the endophytic fungi Esf-14 was prepared by the liquid shaking method. First, some mycelia of the endophytic fungus Esf-14 were selected from cryopreserved tubes stored at 4°C and inoculated on PDA medium plates for 5 to 7 days. After 4 to 5 days of subculture, the mycelia were inoculated in a triangular flask (500 mL) with 200 mL of PDB medium (a total of 100 L), cultured with shaking for 7 days at 25°C and 150 rpm, and then kept static for 21 days. After 1 month, the mycelia and the broth were separated, and the broth was extracted directly with ethyl acetate three times. The mycelia were also extracted with ethyl acetate three times (7 days each time) and further dried and stored at 4°C for further use.

### Isolation of secondary metabolites produced by the endophytic fungi Esf-14.

The EtOAc extract (12.37 g) was subjected to column chromatography (CC) over silica gel (200 to 300 mesh) eluted with a gradient of PE-EtOAc (100:0 to 0:100) to obtain 10 main fractions (F1-8). F2 (1.26 g) was chromatographed over Sephadex LH-20 (eluted with CH_2_Cl_2_-MeOH, 1:1) to afford six subfractions. Subfraction F2-3 (38.9 mg) was further purified by semipreparative high-pressure liquid chromatography (HPLC) (MeOH-H_2_O, 60:40, 4 min/L) to yield compound 2 (1 mg, 19.537 min), and subfraction F2-5 (36 mg) was further purified by semipreparative HPLC (MeOH-H_2_O, 60:40, 4 min/L) to afford compound 1 (6.2 mg, 61.820 min). Similarly, F3 (145.9 mg) was fractionated by CC over Sephadex LH-20 (eluted with CH_2_Cl_2_-MeOH, 1:1), followed by semipreparative HPLC (MeOH-H_2_O, 35:65, 4 min/L) to yield compound 4 (4.1 mg, 32.815 min). F4 (408.6 mg) was separated by chromatography using a Sephadex LH-20 (eluted with CH_2_Cl_2_-MeOH, 1:1) to yield six subfractions. F4-6 (78.8 mg) was directly purified by semipreparative HPLC (MeOH-H_2_O, 50:50, 4 min/L) to afford compound 3 (37.2 mg, 37.083 min). F7 (260.9 mg) was fractionated by CC over Sephadex LH-20 (eluted with CH_2_Cl_2_-MeOH, 1:1) twice, followed by semipreparative HPLC (MeOH-H_2_O, 10:90, 4 min/L) to yield Esf-36-5 compound 5 (2.7 mg, 5.495 min).

### (i) 5,6-Dimethoxy-[1′,1:4,1″-terphenyl]-2-ol (compound 1).

Colorless waxy solid; HR-ESI-MS *m/z* 307.1331 [M+H]^+^ (calculated for C_20_H_19_O_3_, 307.1331), 329.1132 [M+Na]^+^ (calculated for C_23_H_26_O_7_Na, 329.1132); ^1^H NMR (600 MHz, CDCl_3_): δ_H_ 7.59 (m, 2H, H-2″, 6″), 7.53 (m, 2H, H-3′, 5′), 7.44 (m, 5H, H-2′, 4′, 6′, 3′, 5′), 7.36 (m, 1H, H-4″), 6.78 (s, 1H, H-6), 3.68 (s, 3H, OCH_3_-3), 3.60 (s, 3H, OCH_3_-4); ^13^C NMR (151 MHz, CDCl_3_): δ_C_ 151.44 (C-3), 149.13 (C-1), 144.56 (C-4), 138.10 (C-1″), 136.41 (C-5), 132.62 (C-1′), 130.58 and 128.39 (C-2′, 6′, 3′, 5′), 129.46 (C-3′, 5′), 129.34 (C-2″, 6″), 128.50 (C-4′), 127.53 (C-4″), 122.45 (C-2), 112.07 (C-6), 61.17 (3-OCH_3_), 61.04 (4-OCH_3_).

### (ii) 5-(Methoxycarbonyl)-2-methylbenzo[d][1,3]dioxole-2-carboxylic acid (compound 2).

δ_H_ 7.55 (dd, *J *= 1.8, 8.4 Hz, 1H, H-6), 7.34 (d, *J *= 1.2 Hz, 1H, H-4), 6.99 (d, *J *= 7.8 Hz, 1H, H-7), 3.80 (s, 3H, H-9), 1.80 (s, 3H, H-11). δ_C_ 167.36 (C-10), 165.56 (C-8), 151.30 (C-1), 147.49 (C-3), 124.83 (C-6), 123.28 (C-5), 113.57 (C-2), 108.25 (C-4), 107.97 (C-7), 52.01 (C-9), 21.85 (C-11).

### (iii) 3,7-Dihydroxy-1,9-dimethyldibenzofuran (compound 3).

White crystal; ^1^H NMR (600 MHz, acetone-*d*_6_): δ_H_ 8.51 (s, 2H, OH-3, 7), 6.80 (s, 2H, H-4, 6), 6.64 (s, 1H, H-3), 2.80 (s, 6×H, CH_3_-1, 9); ^13^C NMR (151 MHz, acetone-*d*_6_): δ_C_ 158.71 (4a, 5a-C), 156.82 (3, 7-C), 132.81 (1, 9-C), 117.30 (9a, 9b-C), 114.90 (2, 8-C), 96.55 (4, 6-C), 24.98 (1, 9-CH_3_). The ^1^H NMR and ^13^C NMR data were consistent with the literature (48).

### (iv) Methyl 3-hydroxybenzoate (compound 4).

Colorless waxy solid; ^1^H NMR (600 MHz, DMSO): δ_H_ 9.82 (s, 1H, OH-1), 7.38 (dt, *J *= 1.8, 6.0 Hz, 1H, H-6), 7.34 (dd, *J *= 1.8, 2.4 Hz, 1H, H-2), 7.31 (t, *J *= 7.8 Hz, 1H, H-5), 7.02 (m, H-4), 3.82 (s, 3H, CH_3_-8); ^13^C NMR (151 MHz, DMSO): δ_C_ 166.21 (C-7), 157.50 (C-1), 130.82 (C-3), 129.82 (C-5), 120.28 (C-4), 119.76 (C-6), 115.55 (C-2), 52.05 (C-8). The ^1^H NMR and ^13^C NMR data were consistent with the literature (49).

### (v) Uracil (compound 5).

^1^H NMR (600 MHz, DMSO-*d*_6_): δ_H_ 11.00 (br s, H-3), 10.80 (br s, H, H-1), 7.38 (d, 1H, H-6), 5.44 (dd, 1H, H-5); ^13^C NMR (151 MHz, DMSO-*d*_6_): δ_C_ 164.31 (C-4), 151.49 (C-2), 142.17 (C-6), 100.20 (C-5). The ^1^H NMR and ^13^C NMR data were consistent with the literature (50).

### Availability of data and materials.

The generated nucleotide sequence of the endophytic fungal isolates (isolation numbers Cbf-1 to Cbf-11 and Esf-1 to Esf-30) can be accessed in GenBank under accession numbers OK242732 to OK242772 (https://blast.ncbi.nlm.nih.gov/Blast.cgi). The data sets of high-throughput sequencing presented in this study can be found in online repositories. The name of the repository/repositories and accession number can be found at: https://www.ncbi.nlm.nih.gov/, PRJNA952348. All of the fungal isolates were stored in the Laboratory of Plant and Microbial Health, College of Forestry and Landscape Architecture (SCAU). The data sets generated and/or analyzed during the current study are available from the corresponding author on reasonable request.
