# Supplementary material for: Diversity of Fungal Communities on Diseased and Healthy Cinnamomum burmannii Fruits and Antibacterial Activity of Secondary Metabolites
Source: Microbiol Spectr. 2023 May 10;11(3):e00080-23. doi: 10.1128/spectrum.00080-23 (PMC10269519; doi:10.1128/spectrum.00080-23)
Supplement: Supplemental file 1 — Supplemental material. Download spectrum.00080-23-s0001.pdf, PDF file, 2.0 MB [file spectrum.00080-23-s0001.pdf]

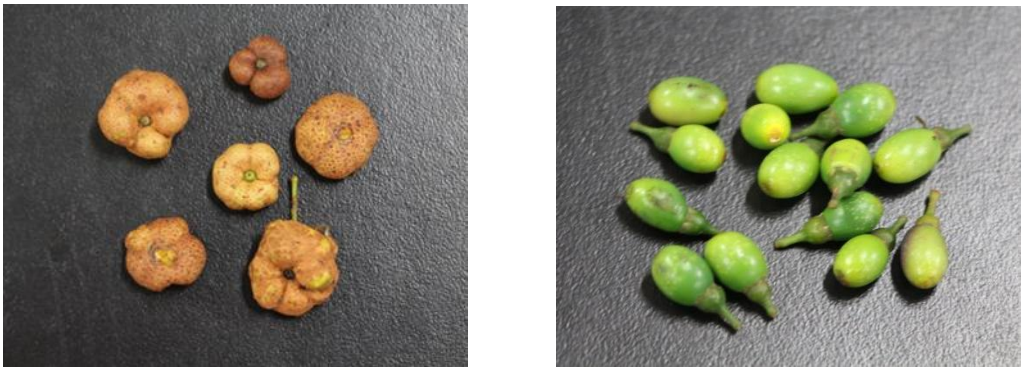

Powdery fruit Healthy fruit

Figure S1 Physical picture of diseased and healthy fruits of *Cinnamomum burmannii*

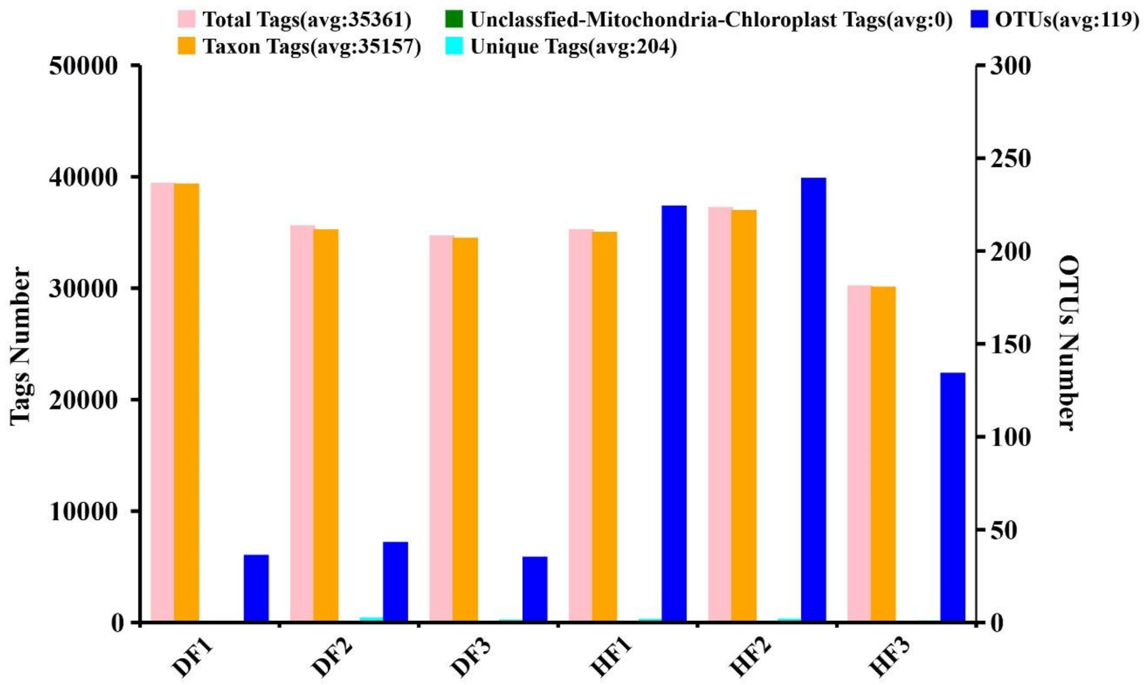

Figure S2 The number of tags and OTUs of fungi in healthy and diseased fruit samples of *C. burmannii.* Total Tags: the total number of stitching sequences obtained after filtering, Taxon Tags: the number of Tags used to build OTUs and obtain classification information, Unclassfied-Mitochondria-Chloroplast Tags: the number of OTU Tags that cannot be annotated to the boundary level, Unique Tags: the number of Tags with frequency 1 that cannot be clustered into OTUs, OTUs: the number of OTUs finally obtained.

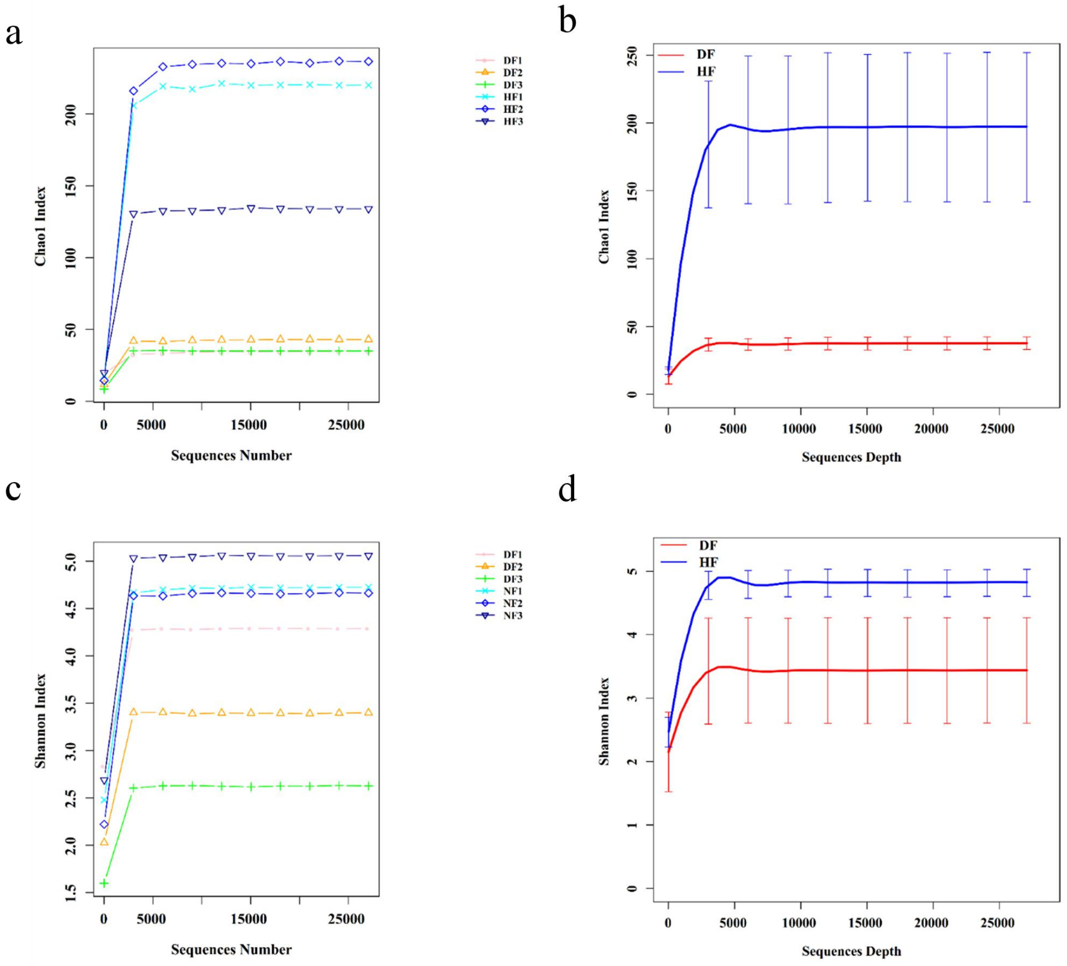

Figure S3 Rarefaction Curve. a is the Chao1 index dilution curve for a single sample, b is the Chao1 index dilution curve between HF and DF groups, c is the Shannon index dilution curve for a single sample, d is the Shannon index dilution curve between HF and DF groups.

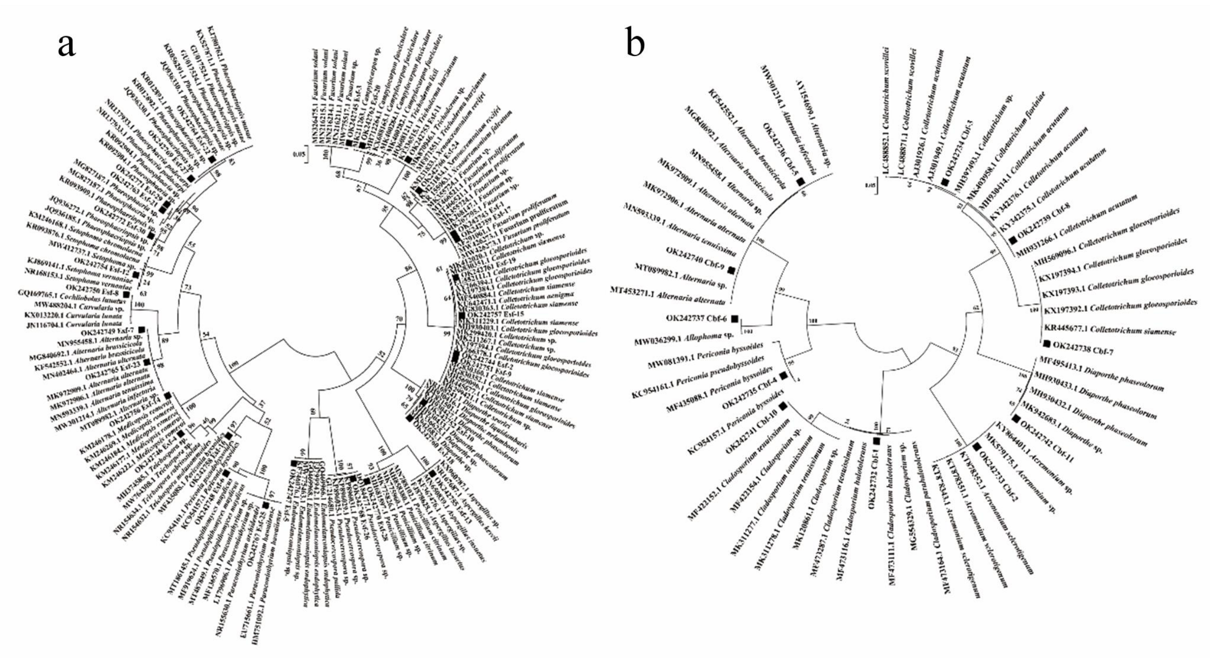

Figure S4 Phylogenetic tree for endophytic fungi from *C. burmannii* based on the rDNA-ITS sequence. a is phylogenetic tree for endophytic fungi of diseased fruit. b is phylogenetic tree for endophytic fungi of healthy fruit.

Table S1 Antibacterial activities forsecondary metabolites of endophytic fungi from *C. burmannii*

Strains	R_f value (inhibition spot diameter)					
	E. coli	*P. lachrymans*	*X. vesicatoria*	*B. subtilis*	*R. solanacearum*	*A. tumefaciens*
Esf-1	0.00-0.47^{+++}	0.00-0.15^{++}; 0.32-0.40^{++}	0.00-0.12^{++}; 0.25-0.37^{++}	0.00-0.42^{+++}	0.00-0.40^{++}	0.00-0.13^{++}; 0.20-0.27^{+}; 0.33-0.37^{++}
Esf-2	---	0.00-0.13^{+}; 0.17-0.27^{++}; 0.30-0.50^{++}	0.00-0.28^{++}	---	0.00-0.48^{++}	0.00-0.13^{++}; 0.17-0.25^{+}; 0.42-0.50^{+}
Esf-3	0.00-0.43^{+++}	0.00-0.25^{+++}	0.00-0.40^{+++}	0.00-0.37^{+++}	0.00-0.38^{+++}	0.02-0.47^{+++}
Esf-4	0.00-0.38^{++}	0.20-0.23^{+}	0.20-0.23^{+}	0.18-0.45^{+++}	0.00-0.48^{++}	-
Esf-5	0.00-0.50^{++}	0.00-0.25^{++}; 0.32-0.47^{++}	0.00-0.43^{++}	0.00-0.43^{++}	0.00-0.42^{++}	0.00-0.05^{+}; 0.32-0.42^{+}
Esf-6	0.03-0.08^{+} 0.22-0.30^{++}	0.00-0.52^{++}	0.00-0.37^{++}	0.00-0.12^{+}; 0.18-0.25^{++}; 0.35-0.40^{+}	0.00-0.50^{++}	0.20-0.25^{+}
Esf-7	0.00-0.10^{+}; 0.25-0.27^{+}	0.00-0.30^{++}; 0.43-0.47^{+}	0.00-0.17^{++}; 0.22-0.27^{+}	0.00-0.12^{++}; 0.18-0.23^{+}	0.00-0.28^{++}; 0.43-0.50^{++}	0.00-0.17^{+}
Esf-8	0.00-0.55^{++}	0.00-0.22^{++}; 0.25-0.33^{++}; 0.50-0.55^{++}	0.00-0.60^{+++}	0.00-0.52^{+++}	0.00-0.43^{++}	0.00-0.08^{+}; 0.23-0.28^{+}; 0.37-0.47^{++}
Esf-9	0.00-0.50^{+++}	0.00-0.33^{++}; 0.38-0.45^{++}; 0.47-0.57^{+}	0.00-0.60^{+++}	0.00-0.48^{+++}	0.00-0.38^{+++}	0.00-0.17^{+}; 0.22-0.25^{+}; 0.33-0.45^{++}
Esf-10	---	---	0.00-0.18^{++}; 0.23-0.35^{++}	0.37-0.42^{+}	---	0.00-0.07^{+}; 0.23-0.27^{+}; 0.42-0.50^{+}
Esf-11	0.00-0.08^{++}; 0.33-0.42^{+}	0.00-0.12^{++}	0.00-0.30^{++}; 0.37-0.50^{++}	0.00-0.13^{++}; 0.38-0.45^{++}	0.00-0.13^{++}; 0.42-0.45^{+}	0.00-0.18^{++}; 0.22-0.25^{++}; 0.32-0.50^{++}
Esf-12	0.08-0.15^{+}	---	0.00-0.20^{++}; 0.23-0.32^{++}; 0.33-0.47^{+}	0.00-0.05^{+}; 0.10-0.17^{+}	0.00-0.07^{+}; 0.45-0.48^{+}	0.00-0.17^{++}; 0.22-0.25^{+}; 0.35-0.45^{+}
Esf-13	0.00-0.05^{+}	0.22-0.27^{+}	0.00-0.13^{++}; 0.20-0.37^{++}; 0.45-0.48^{+}	0.00-0.07^{+}; 0.23-0.28^{+}	0.23-0.30^{+}	0.18-0.27^{++}; 0.33-0.42^{+}
Esf-14	0.00-0.03^{+}; 0.17-0.05^{+};	0.00-0.08^{+}; 0.17-0.28^{++};	0.00-0.13^{+}; 0.17-0.37^{++};	0.00-0.53^{++}	0.00-0.10^{+}; 0.12-0.30^{++};	0.02-0.05^{+}; 0.07-0.13^{+};

	0.40-0.43^{+}	0.42-0.50^{++}	0.38-0.55^{++}		0.33-0.48^{++}	0.18-0.37^{+}
Esf-15	0.00-0.17^{++}; 0.22-0.28^{++}; 0.30-0.35^{+}; 0.43-0.48^{+}	0.00-0.13^{++}; 0.25-0.30^{++}	0.00-0.57^{++}	0.00-0.50^{++}	0.00-0.40^{++}	0.00-0.10^{+++}
Esf-16	0.00-0.03^{+}	0.00-0.10^{++}; 0.20-0.25^{+}; 0.38-0.47^{++}	0.00-0.55^{++}	0.00-0.53^{+++}	0.00-0.30^{++}; 0.32-0.52^{++}	0.35-0.38^{+}
Esf-17	0.00-0.07^{++}	0.00-0.10^{++}; 0.22-0.23^{+}; 0.38-0.42^{+}	0.00-0.20^{++}; 0.22-0.30^{+}; 0.38-0.50^{++}	0.00-0.47^{+++}	0.00-0.13^{++}; 0.18-0.27^{+}; 0.35-0.47^{++}	0.08-0.15^{++}
Esf-18	0.00-0.13^{++}; 0.20-0.33^{+}	0.00-0.37^{++}	0.00-0.43^{++}; 0.47-0.53^{++}	0.00-0.48^{+++}; 0.50-0.53^{+}	0.00-0.43^{++}; 0.45-0.50^{++}	0.37-0.42^{+}
Esf-19	0.00-0.13^{++}; 0.18-0.30^{++}; 0.37-0.50^{++}	0.00-0.40^{++}	0.00-0.57^{++}	0.00-0.52^{+++}	0.00-0.53^{+++}	0.00-0.10^{++}
Esf-20	0.00-0.07^{+}	0.00-0.17^{++}; 0.22-0.27^{+}; 0.37-0.42^{++}	0.00-0.08^{+}; 0.13-0.17^{+}; 0.35-0.48^{+}	0.00-0.47^{++}	0.00-0.17^{++}; 0.22-0.28^{+}; 0.33-0.43^{++}	0.20-0.25^{+}; 0.35-0.40^{+}
Esf-21	0.00-0.28^{++}	0.00-0.08^{++}; 0.17-0.23^{++}; 0.35-0.40^{+}	0.00-0.08^{++}; 0.17-0.27^{++}; 0.40-0.45^{++}	0.00-0.12^{++}; 0.18-0.47^{++}	0.00-0.12^{++}; 0.22-0.33^{++}; 0.37-0.53^{++}	0.00-0.07^{++}; 0.17-0.23^{++}; 0.38-0.42^{+}
Esf-22	0.00-0.05^{++}	0.02-0.50^{+++}	0.00-0.55^{+++}	0.00-0.50^{+++}	0.00-0.38^{+++}	0.00-0.10^{+}; 0.23-0.33^{++}
Esf-23	0.00-0.17^{++}	0.00-0.17^{++}; 0.22-2.67^{+}; 0.38-0.50^{++}	0.00-0.30^{++}; 0.37-0.52^{++}	0.00-0.50^{++}	0.00-0.22^{++}; 0.25-0.38^{++}	0.00-0.12^{++}; 0.13-0.18^{+}
Esf-24	0.00-0.05^{+}	0.00-0.17^{++}; 0.20-0.23^{+}; 0.33-0.52^{++}	0.00-0.18^{++}; 0.18-0.25^{+}; 0.30-0.52^{+++}	0.00-0.50^{+++}	0.00-0.40^{+++}	0.00-0.08^{++}
Esf-25	---	0.00-0.15^{+}; 0.37-0.47^{++}	0.00-0.08^{+}; 0.10-0.18^{+}; 0.32-0.47^{++}	0.00-0.25^{++}; 0.18-0.43^{++}	0.00-0.17^{++}; 0.20-0.37^{++}	0.00-0.40^{++}; 0.43-0.50^{+}
Esf-26	---	0.00-0.15^{++}; 0.22-0.25^{+}; 0.35-0.48^{++}	0.00-0.28^{++}; 0.33-0.48^{+++}	0.00-0.43^{+++}	0.00-0.12^{++}; 0.13-0.33^{+++}	0.00-0.53^{++}
Esf-27	---	0.00-0.05^{+}; 0.13-0.25^{++}; 0.38-0.47^{+}	0.13-0.32^{+}; 0.38-0.52^{++}	0.00-0.08^{+}; 0.15-0.40^{++}	0.00-0.38^{++}	0.00-0.20^{++}; 0.30-0.45^{++}
Esf-28	---	0.00-0.15^{++}; 0.22-0.25^{+}; 0.35-0.48^{++}	0.00-0.28^{++}; 0.33-0.48^{+++}	0.00-0.43^{+++}	0.00-0.12^{++}; 0.13-0.33^{+++}	0.00-0.53^{++}

Esf-29	0.00-0.08^{+}<br>0.23-0.28^{+}<br>0.43-0.48^{+}	0.00-0.08^{+}	0.00-0.12^{+}<br>0.25-0.30^{++}<br>0.43-0.48^{+}	0.00-0.28^{++}<br>0.33-0.50^{++}	0.00-0.55^{+++}	0.00-0.10^{+}<br>0.4-0.48^{+}
Esf-30	0.00-0.07^{+};<br>0.10-0.17^{++}	0.00-0.07^{+};<br>0.08-0.15^{++}	0.00-0.17^{++}	0.00-0.07^{+};<br>0.08-0.17^{++}	0.00-0.45^{++}	0.00-0.15^{++}
Cbf-1	0.00-0.05^{+};<br>0.17-0.25^{+};<br>0.33-0.50^{++}	-	0.00-0.25^{++};<br>0.40-0.47^{+}	0.42-0.47^{+}	0.00-0.25^{++};<br>0.32-0.50^{++}	0.00-0.05^{+};<br>0.38-0.42^{+}
Cbf-2	0.00-0.50^{++}	0.00-0.15^{++};<br>0.18-0.25^{+};<br>0.35-0.43^{+}	0.00-0.52^{++}	0.00-0.23^{++};<br>0.35-0.45^{+}	0.00-0.25^{++};<br>0.33-0.50^{+}	0.00-0.10^{+};<br>0.15-0.22^{+};<br>0.32-0.40^{+}
Cbf-3	0.40-0.48^{+}	0.00-0.07^{+}	0.00-0.10^{+};<br>0.27-0.28^{+};<br>0.48-0.53^{++}	-	-	0.03-0.08^{+}
Cbf-4	0.00-0.33^{++};<br>0.37-0.50^{+}	0.10-0.13^{+};<br>0.20-0.27^{+}	0.10-0.13^{+};<br>0.20-0.27^{++}	0.20-0.27^{++};<br>0.32-0.40^{++}	0.00-0.30^{++};<br>0.33-0.40^{+};<br>0.42-0.50^{++}	-
Cbf-5	0.00-0.10^{+}<br>0.20-0.23^{+}	-	0.00-0.10^{+}	0.40-0.48^{+}	0.00-0.22^{+}	0.37-0.45^{+}
Cbf-6	0.00-0.18^{++};<br>0.22-0.28^{+};<br>0.37-0.55^{++}	0.07-0.15^{++}	0.07-0.13^{++}	0.02-0.17^{++};<br>0.20-0.28^{++}; 0.32-0.5^{++}	0.00-0.27^{++};<br>0.42-0.50^{++}	0.05-0.12^{+};<br>0.37-0.43^{+}
Cbf-7	0.00-0.15^{+};<br>0.25-0.40^{++};<br>0.42-0.58^{+}	0.22-0.25^{+};<br>0.37-0.47^{+}	0.00-0.08^{+};<br>0.20-0.25^{+}	0.00-0.48^{+++}	0.00-0.50^{++}	0.00-0.10^{+}
Cbf-8	0.00-0.10^{+}	0.38-0.47^{+}	0.37-0.47^{++}	0.00-0.52^{++}	-	0.02-0.07^{+}<br>0.33-0.43^{++}
Cbf-9	-	0.00-0.12^{+};<br>0.23-0.33^{++}	0.00-0.35^{++};<br>0.45-0.52^{+}	0.03-0.12^{+};<br>0.20-0.32^{++}	0.00-0.12^{+};<br>0.22-0.55^{++}	0.00-0.10^{+};<br>0.20-0.32^{++};<br>0.40-0.45^{+}
Cbf-10	0.23-0.28^{+}	0.00-0.08^{+};<br>0.23-0.28^{+}	0.00-0.18^{+};<br>0.22-0.33^{++};<br>0.40-0.58^{++}	0.03-0.07^{+};<br>0.17-0.35^{++};<br>0.42-0.53^{++}	0.00-0.13^{+};<br>0.25-0.30^{++};<br>0.45-0.60^{++}	0.00-0.07^{+};<br>0.22-0.25^{++};<br>0.42-0.50^{++}
Cbf-11	0.23-0.28^{+}	0.00-0.050^{+}	0.00-0.10^{+};<br>0.18-0.33^{++};<br>0.38-0.47^{+}	0.18-0.45^{++}	0.00-0.08^{++};<br>0.13-0.52^{++}	0.00-0.07^{+}
Streptomycin sulfate	++	++	++	++	++	++

---: Inhibition spot was not observed; +: Maximum inhibition spot diameter d < 5 mm; ++: Maximum inhibition spot diameter 5 mm≤d < 10 mm; +++: Maximum inhibition spot diameter d ≥10 mm; The positive control streptomycin sulfate was only sampled on TLC plate.

Acquisition Parameter

Source Type	ESI	Ion Polarity	Positive	Set Nebulizer	0.4 Bar
Focus	Active	Set Capillary	4500 V	Set Dry Heater	180 °C
Scan Begin	100 m/z	Set End Plate Offset	-500 V	Set Dry Gas	4.0 l/min
Scan End	1500 m/z	Set Charging Voltage	0 V	Set Divert Valve	Waste
		Set Corona	0 nA	Set APCI Heater	0 °C

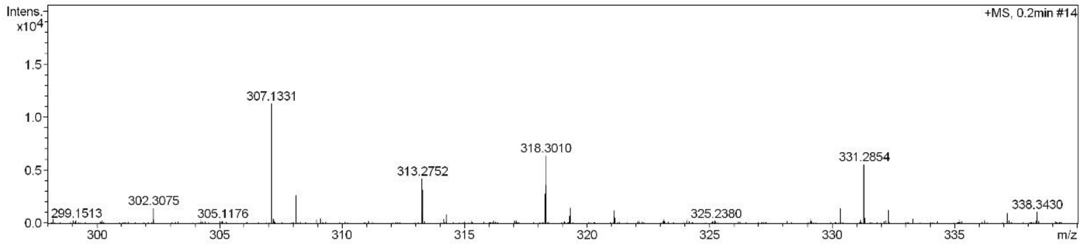

Meas. m/z	#	Ion Formula	Score	m/z	err [ppm]	err [mDa]	mSigma	rdb	e⁻ Conf	N-Rule
307.1331	1	C20H19O3	100.00	307.1329	0.7	0.2	10.1	11.5	even	ok
329.1132	1	C20H18NaO3	100.00	329.1148	-5.0	-1.6	223.7	11.5	even	ok

Figure S5 HR-ESI-MS spectrum of 5, 6-dimethoxy-[1',1:4,1''-terphenyl]-2-ol (**1**)

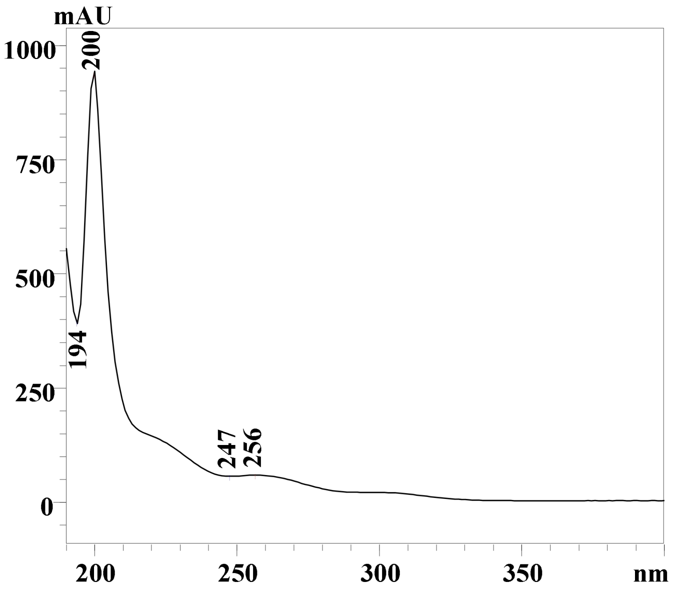

Figure S6 UV spectrum of 5, 6-dimethoxy-[1',1:4,1''-terphenyl]-2-ol (**1**)

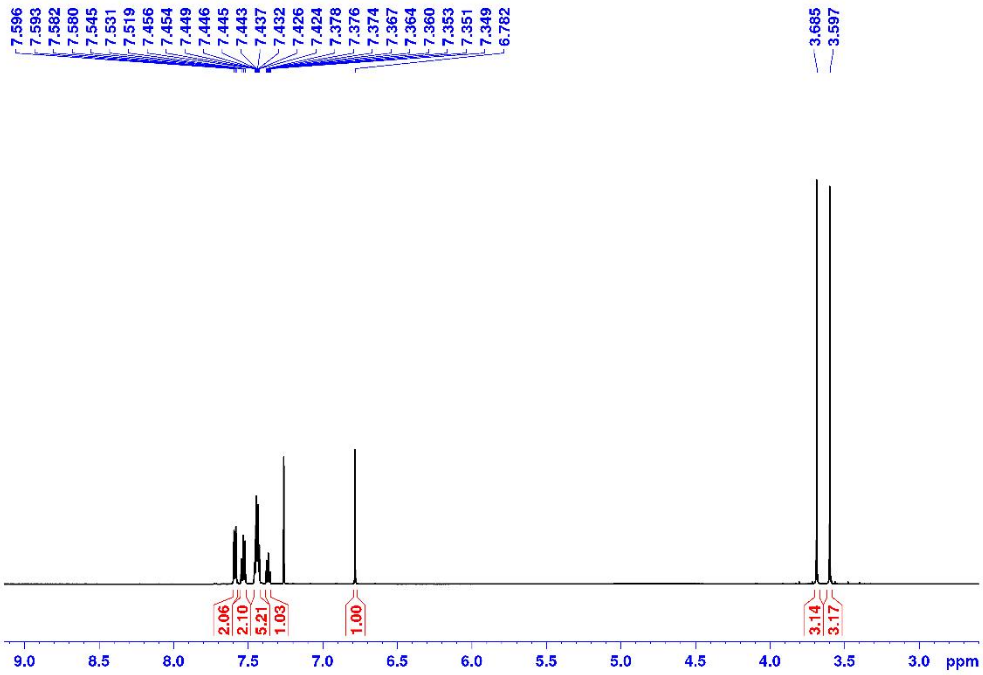

Figure S7 ^{1}H NMR spectrum of 5, 6-dimethoxy-[1',1:4,1''-terphenyl]-2-ol (**1**) ($CDCl_3$, 600 MHz)

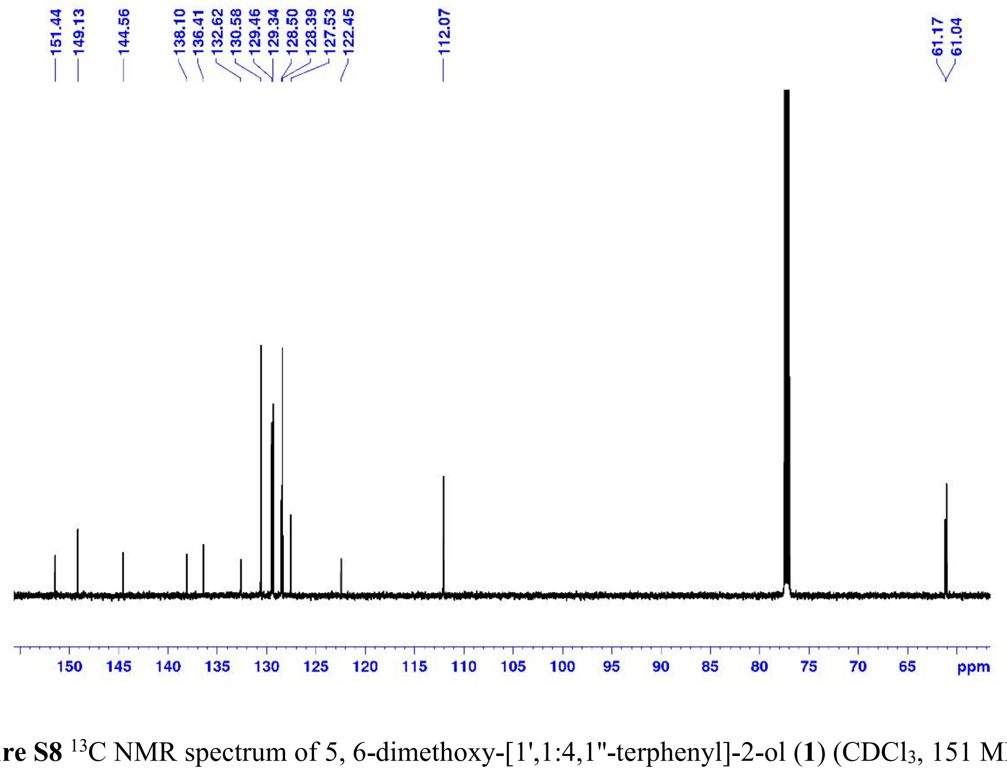

Figure S8 ^{13}C NMR spectrum of 5, 6-dimethoxy-[1',1:4,1''-terphenyl]-2-ol (**1**) ($CDCl_3$, 151 MHz)

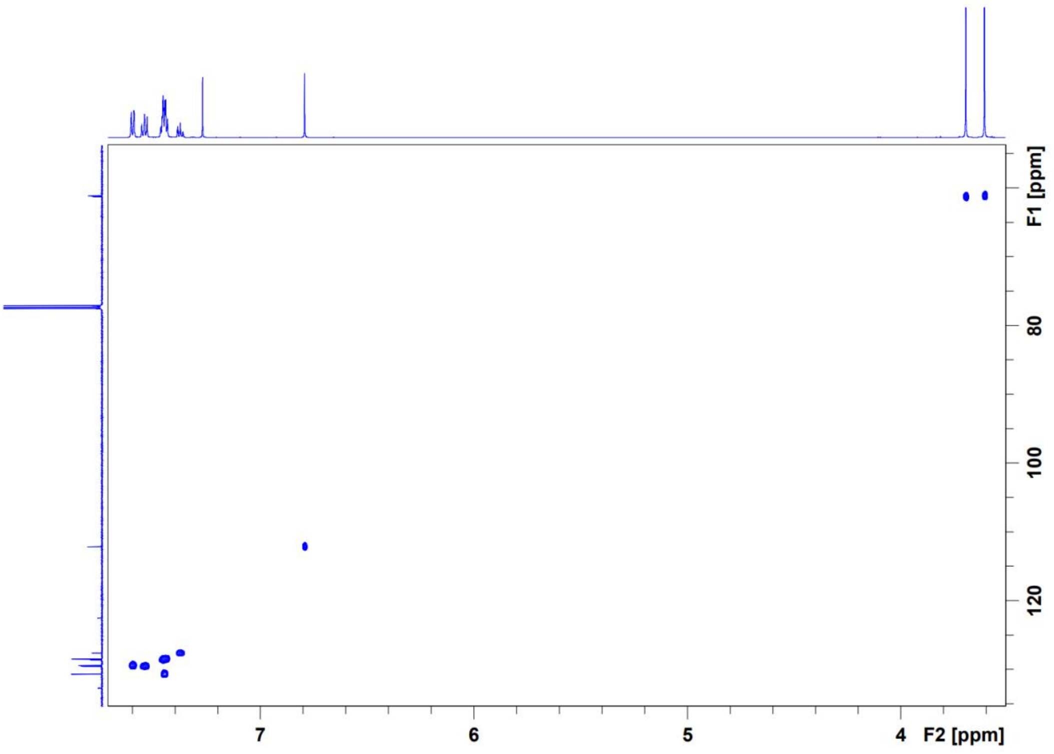

Figure S9 HSQC spectrum of 5, 6-dimethoxy-[1',1:4,1''-terphenyl]-2-ol (**1**) ($CDCl_3$)

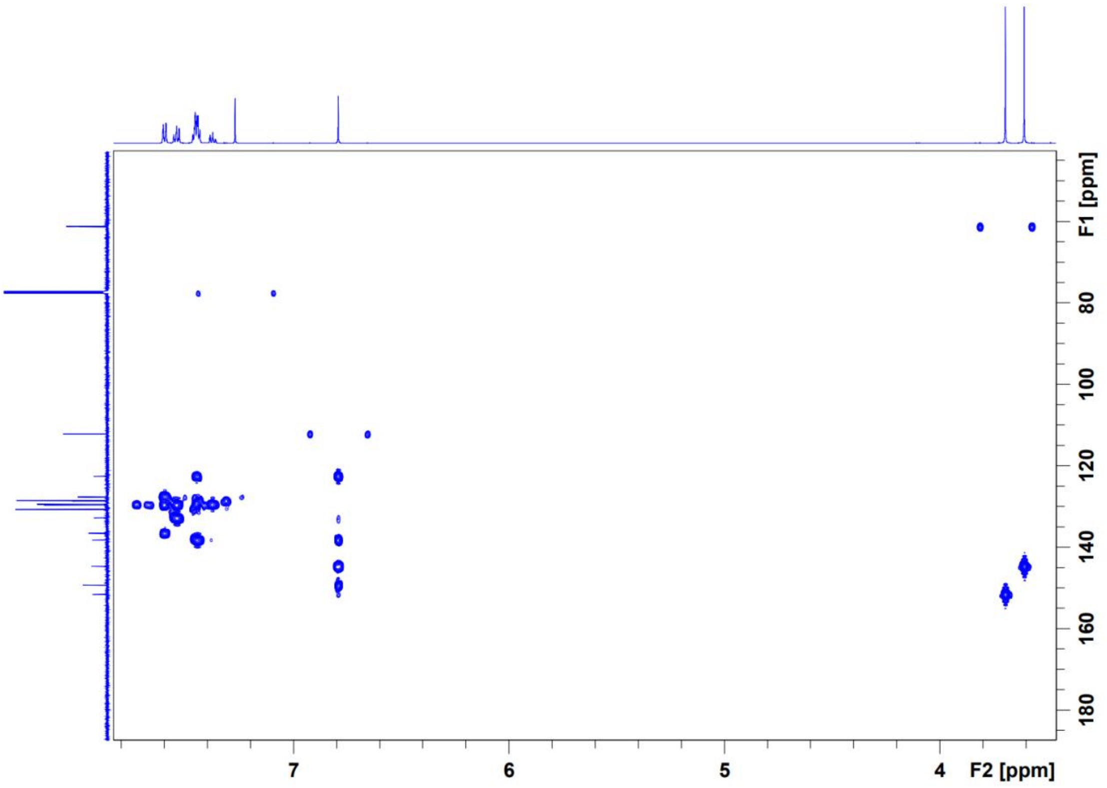

Figure S10 HMBC spectrum of 5, 6-dimethoxy-[1',1:4,1''-terphenyl]-2-ol (**1**) ($CDCl_3$)

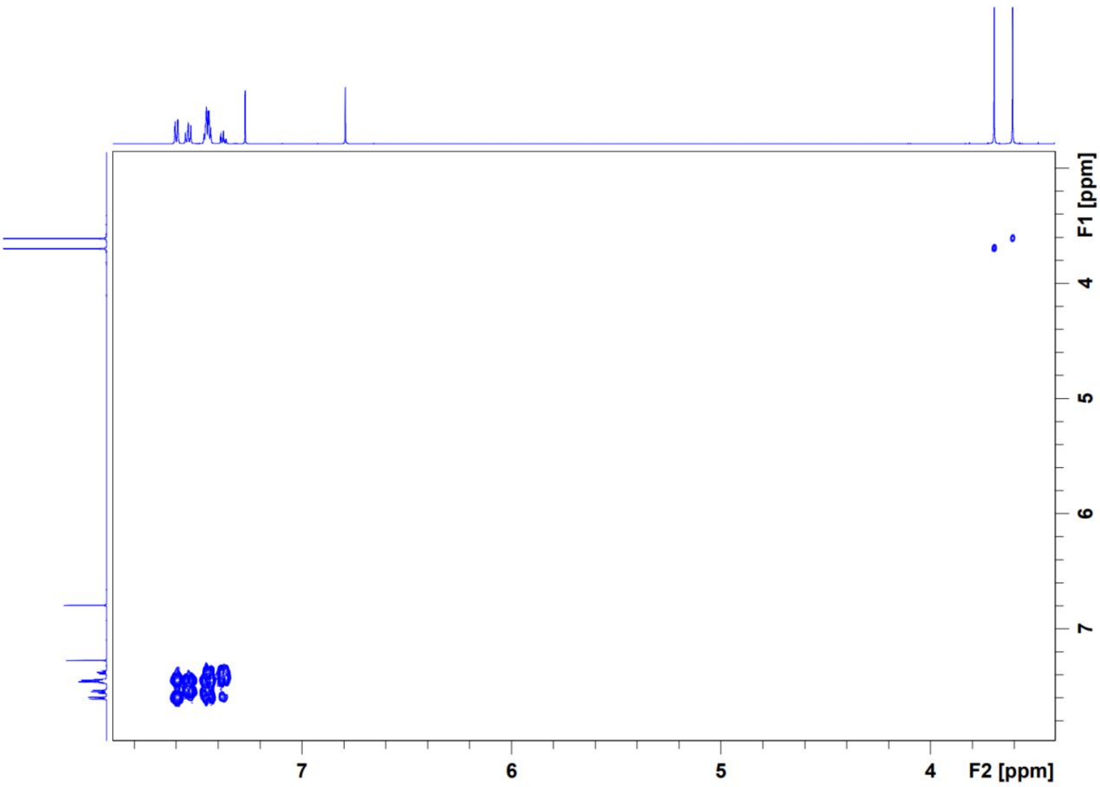

Figure S11 ^{1}H-^{1}H COSY spectrum of 5, 6-dimethoxy-[1',1:4,1''-terphenyl]-2-ol (**1**) ($CDCl_3$)

Acquisition Parameter

Source Type	ESI	Ion Polarity	Negative	Set Nebulizer	0.4 Bar
Focus	Active	Set Capillary	4200 V	Set Dry Heater	180 °C
Scan Begin	100 m/z	Set End Plate Offset	-500 V	Set Dry Gas	4.0 l/min
Scan End	1500 m/z	Set Charging Voltage	0 V	Set Divert Valve	Waste
		Set Corona	0 nA	Set APCI Heater	0 °C

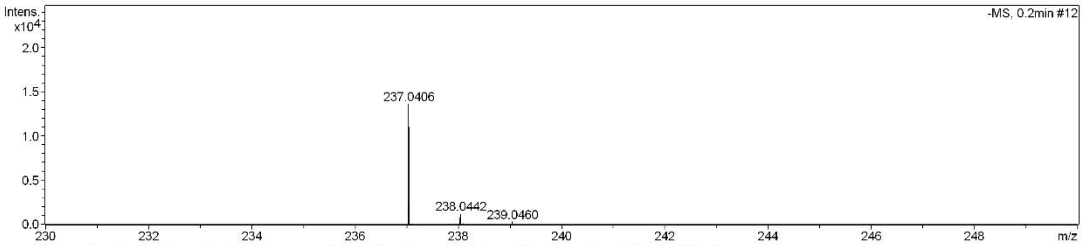

Meas. m/z	#	Ion Formula	Score	m/z	err [ppm]	err [mDa]	mSigma	rdb	e⁻ Conf	N-Rule
237.0406	1	C11H9O6	100.00	237.0405	0.8	0.2	19.4	7.5	even	ok
475.0881	1	C22H19O12	100.00	475.0882	0.2	0.1	31.4	13.5	even	ok

Figure S12 HR-ESI-MS spectrum of 5-(methoxycarbonyl)-2-methylbenzo[d][1, 3]dioxole-2-carboxylic acid (**2**)

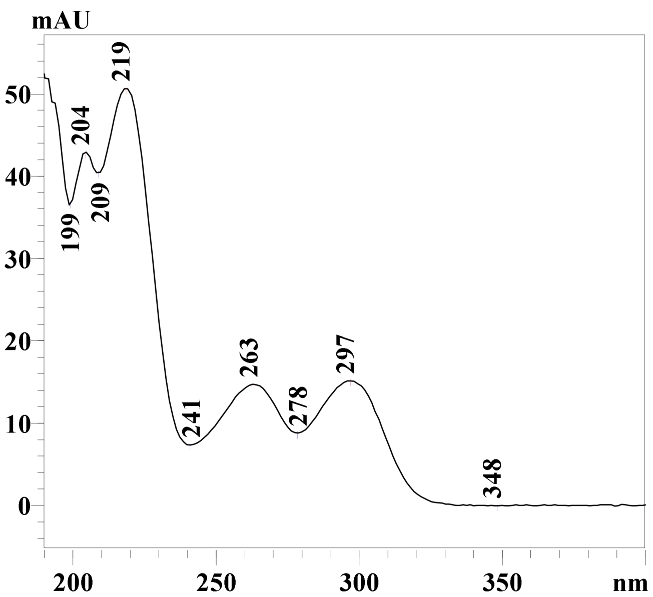

Figure S13 UV spectrum of 5-(methoxycarbonyl)-2-methylbenzo[d][1, 3]dioxole-2-carboxylic acid (**2**)

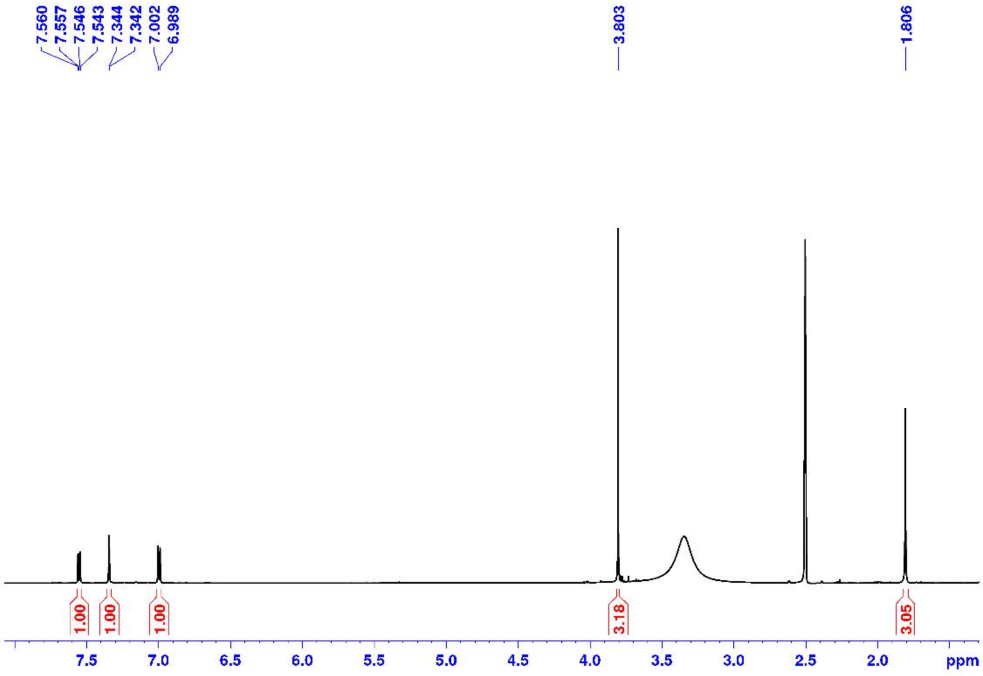

Figure S14 ^{1}H NMR spectrum of 5-(methoxycarbonyl)-2-methylbenzo[d][1, 3]dioxole-2-carboxylic acid (**2**) (DMSO-d_6, 600 MHz)

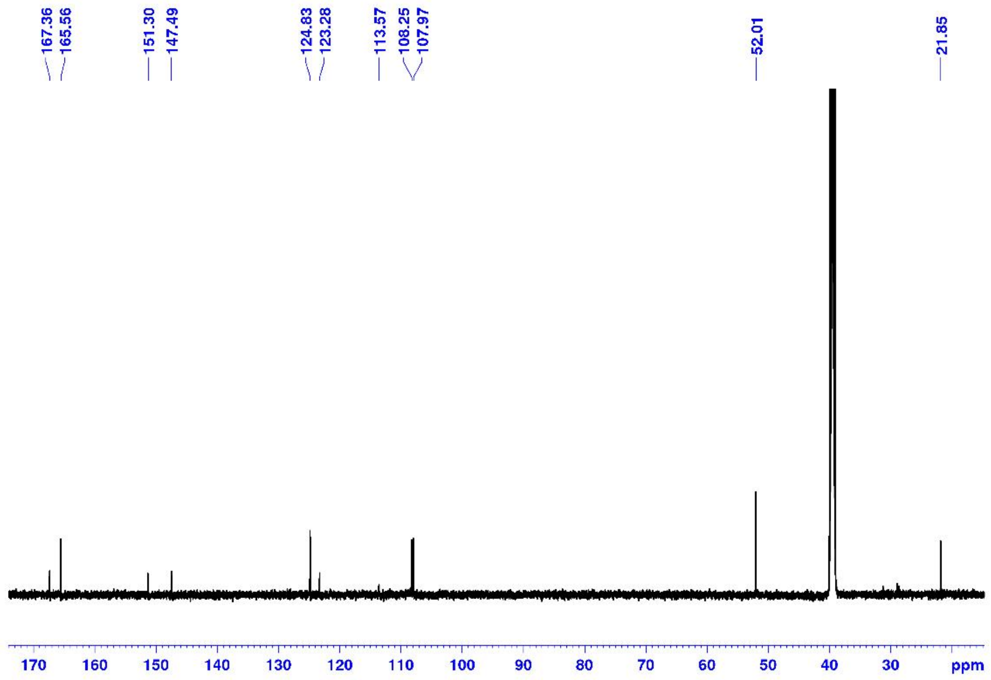

Figure S15 ^{13}C NMR spectrum of 5-(methoxycarbonyl)-2-methylbenzo[d][1, 3]dioxole-2-carboxylic acid (**2**) (DMSO-d_6, 151 MHz)

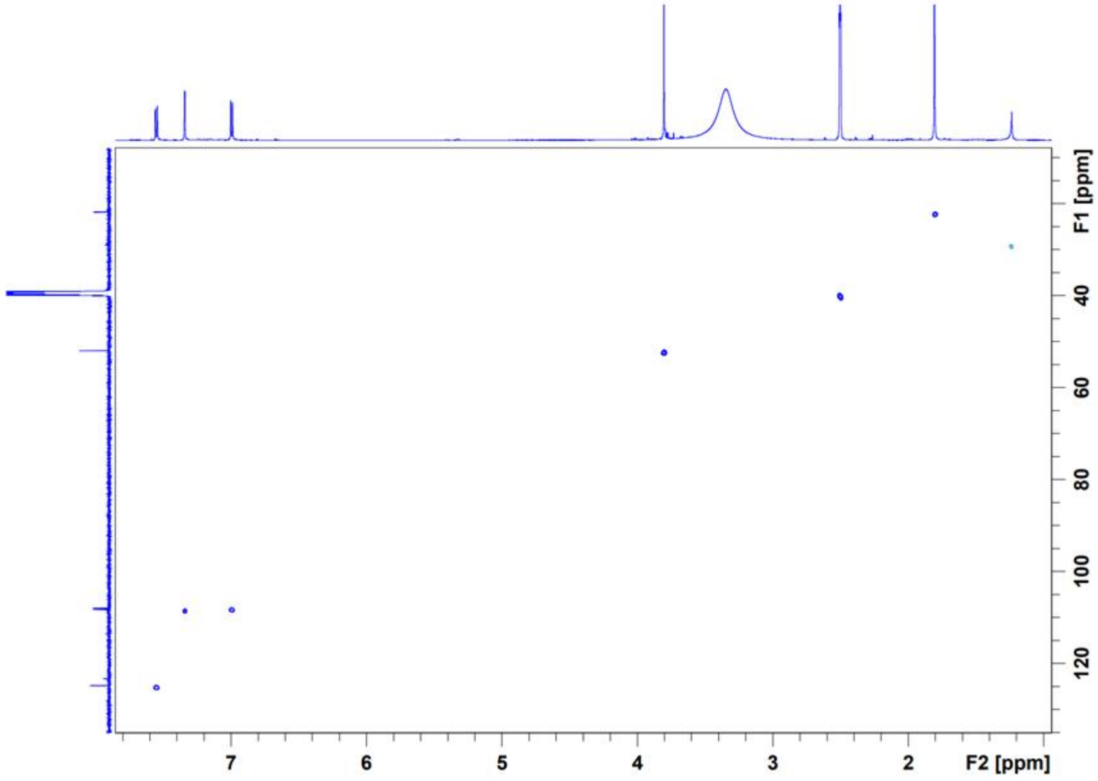

Figure S16 HSQC spectrum of 5-(methoxycarbonyl)-2-methylbenzo[d][1, 3]dioxole-2-carboxylic acid (**2**) (DMSO-d_6)

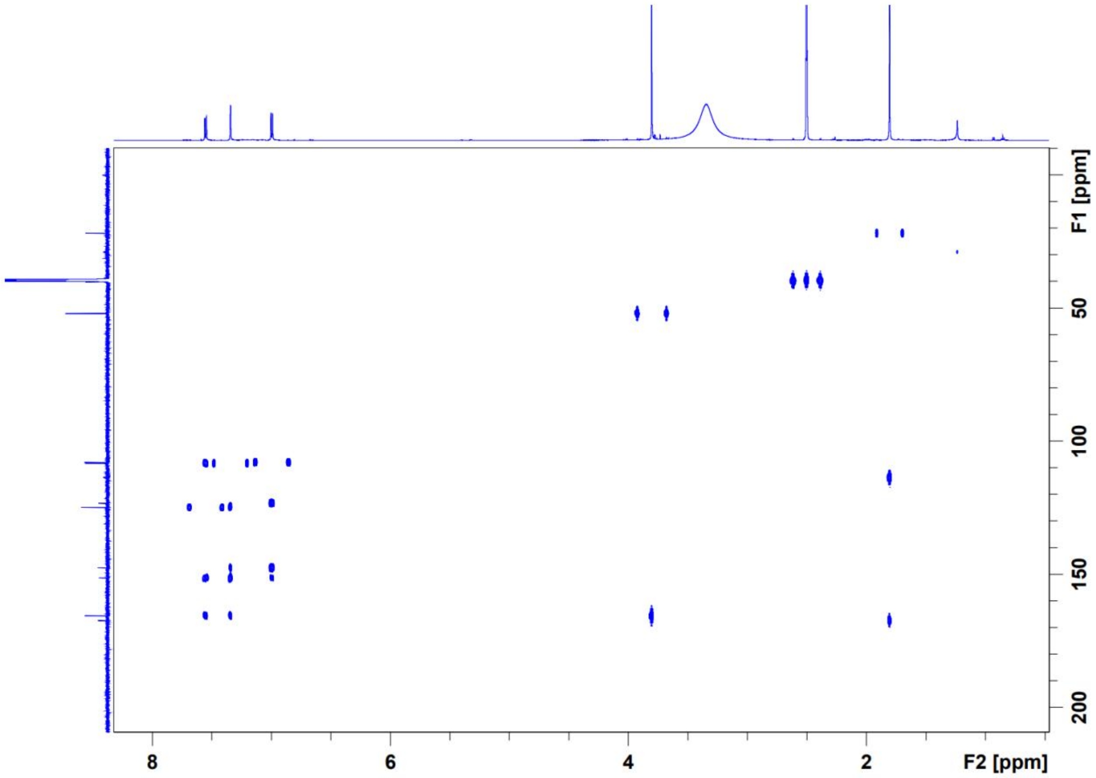

Figure S17 HMBC spectrum of 5-(methoxycarbonyl)-2-methylbenzo[d][1, 3]dioxole-2-carboxylic acid (**2**) (DMSO-d_6)

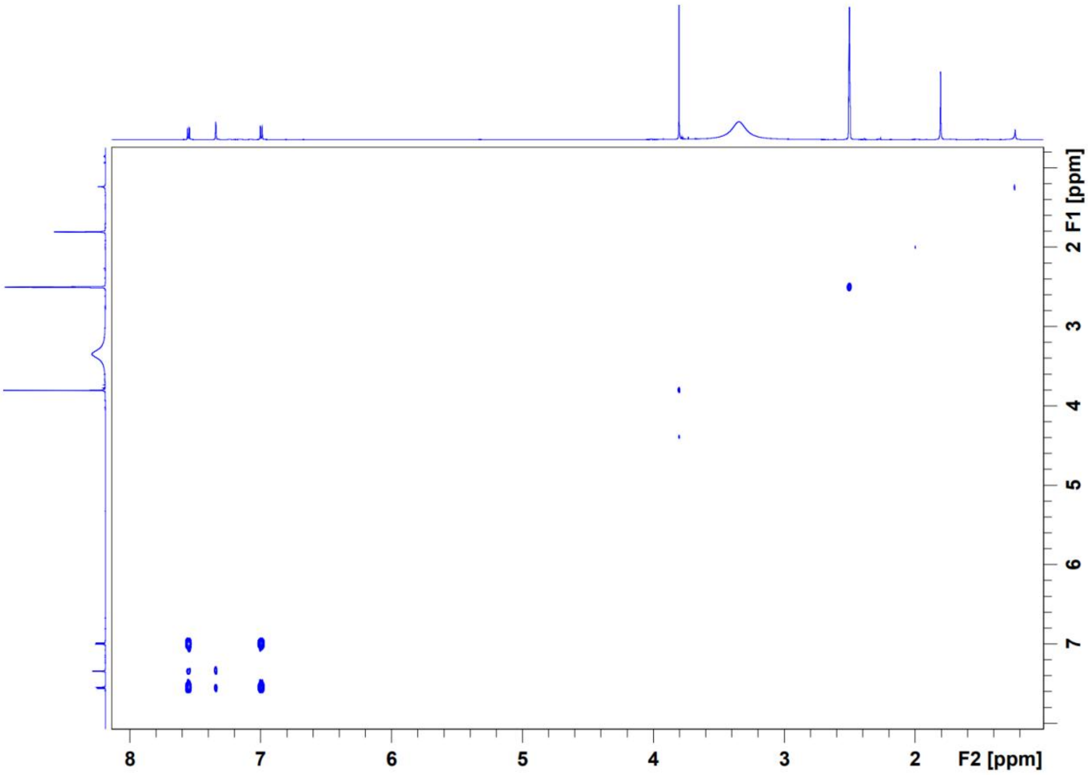

Figure S18 ^1H-^1H COSY spectrum of 5-(methoxycarbonyl)-2-methylbenzo[d][1, 3]dioxole-2-carboxylic acid (**2**) (DMSO-d_6)

Acquisition Parameter

Source Type	ESI	Ion Polarity	Positive	Set Nebulizer	0.4 Bar
Focus	Active	Set Capillary	4500 V	Set Dry Heater	180 °C
Scan Begin	70 m/z	Set End Plate Offset	-500 V	Set Dry Gas	4.0 l/min
Scan End	1500 m/z	Set Charging Voltage	0 V	Set Divert Valve	Waste
		Set Corona	0 nA	Set APCI Heater	0 °C

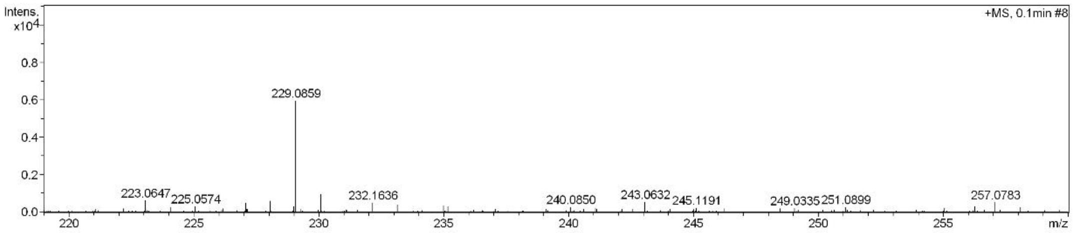

Meas. m/z	#	Ion Formula	Score	m/z	err [ppm]	err [mDa]	mSigma	rdb	e⁻ Conf	N-Rule
229.0859	1	C14H13O3	100.00	229.0859	0.2	0.0	6.9	8.5	even	ok

Figure S19 HR-ESI-MS spectrum of 3,7-dihydroxy-1,9-dimethyldibenzofuran (**3**)

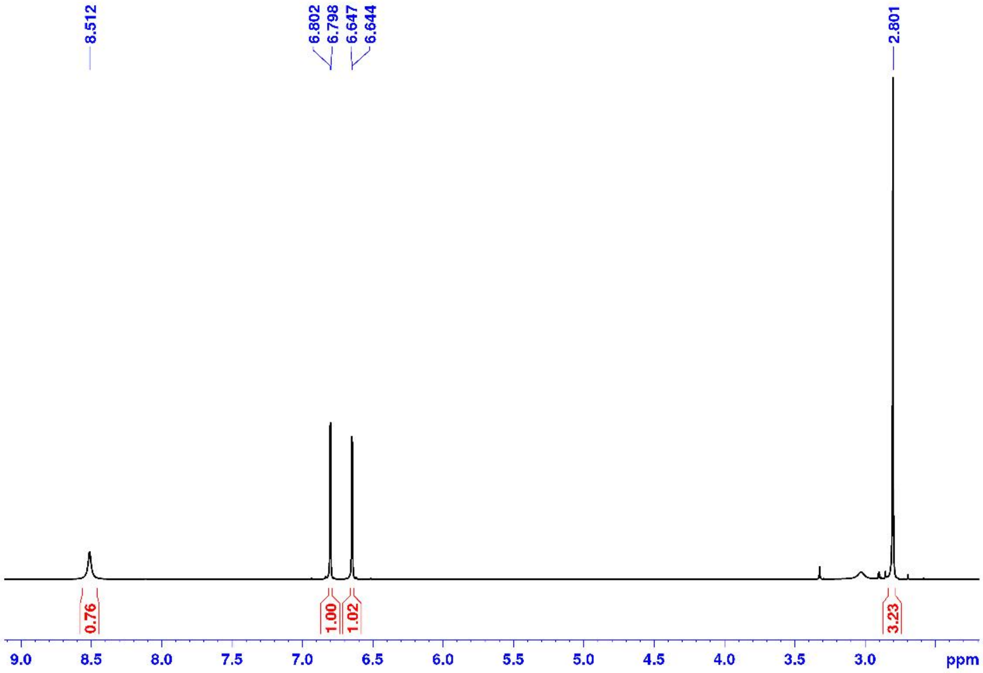

Figure S20 ^{1}H NMR spectrum of 3,7-dihydroxy-1,9-dimethyldibenzofuran (**3**) (Acetone-d_6, 600 MHz)

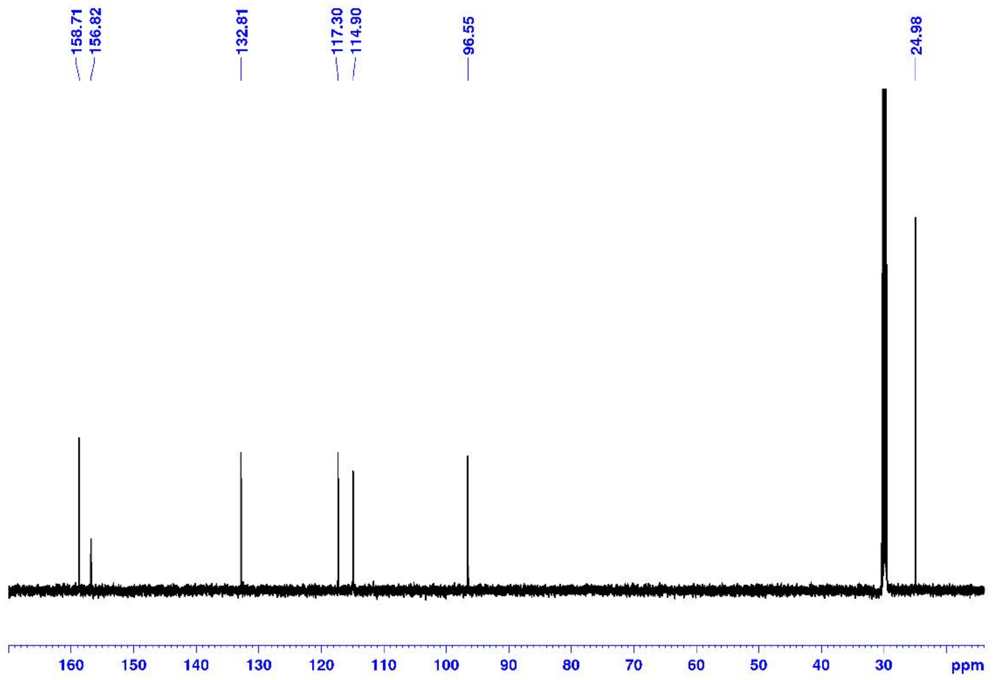

Figure S21 ^{13}C NMR spectrum of 3,7-dihydroxy-1,9-dimethyldibenzofuran (**3**) (Acetone-d_6, 151 MHz)

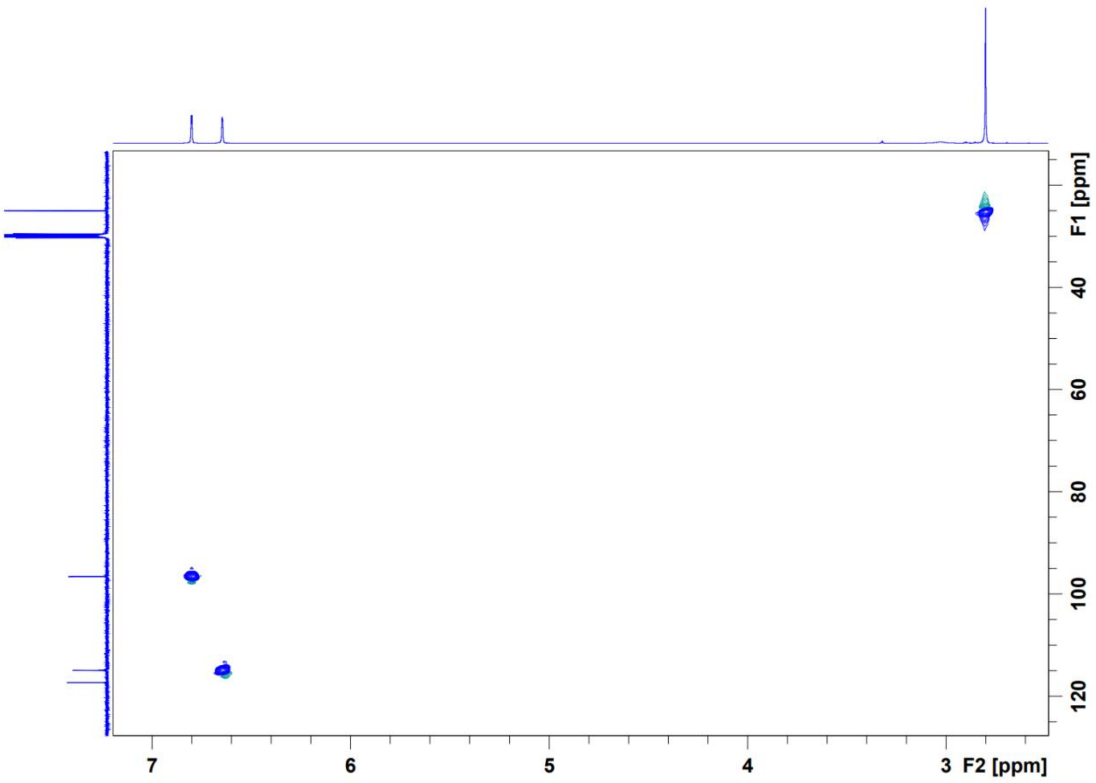

Figure S22 HSQC spectrum of 3,7-dihydroxy-1,9-dimethyldibenzofuran (**3**) (Acetone-d_6)

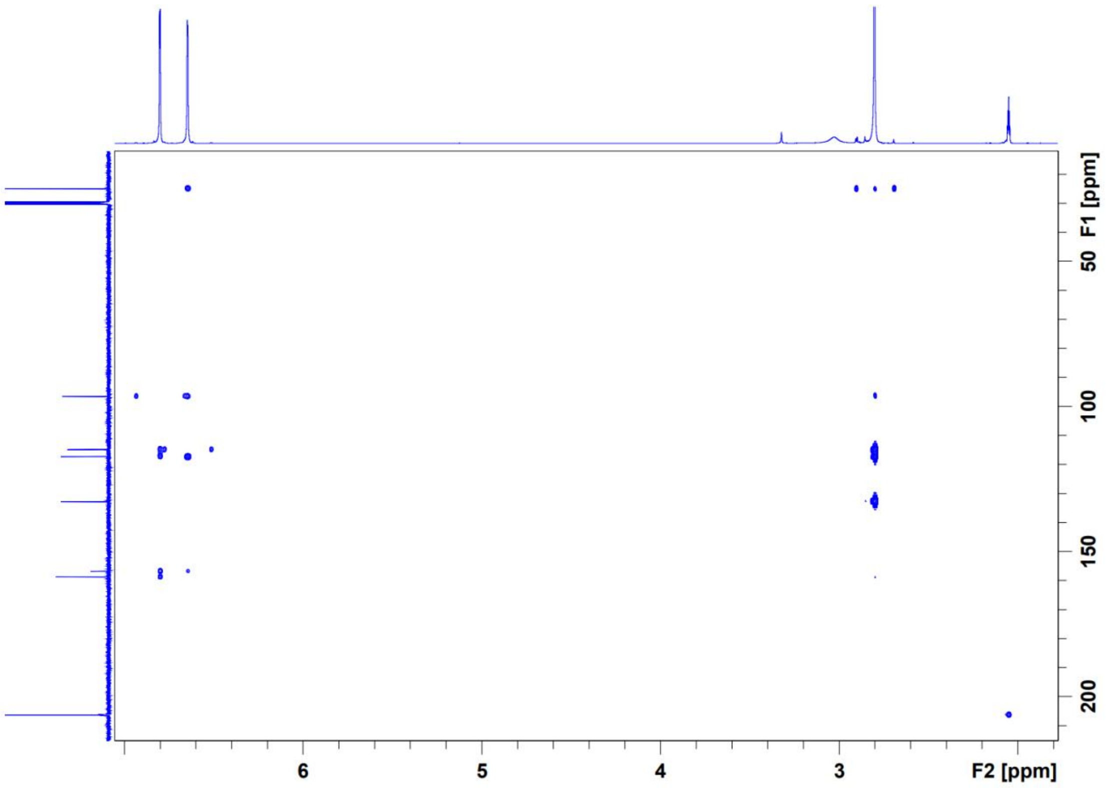

Figure S23 HMBC spectrum of 3,7-dihydroxy-1,9-dimethyldibenzofuran (**3**) (Acetone-d_6)

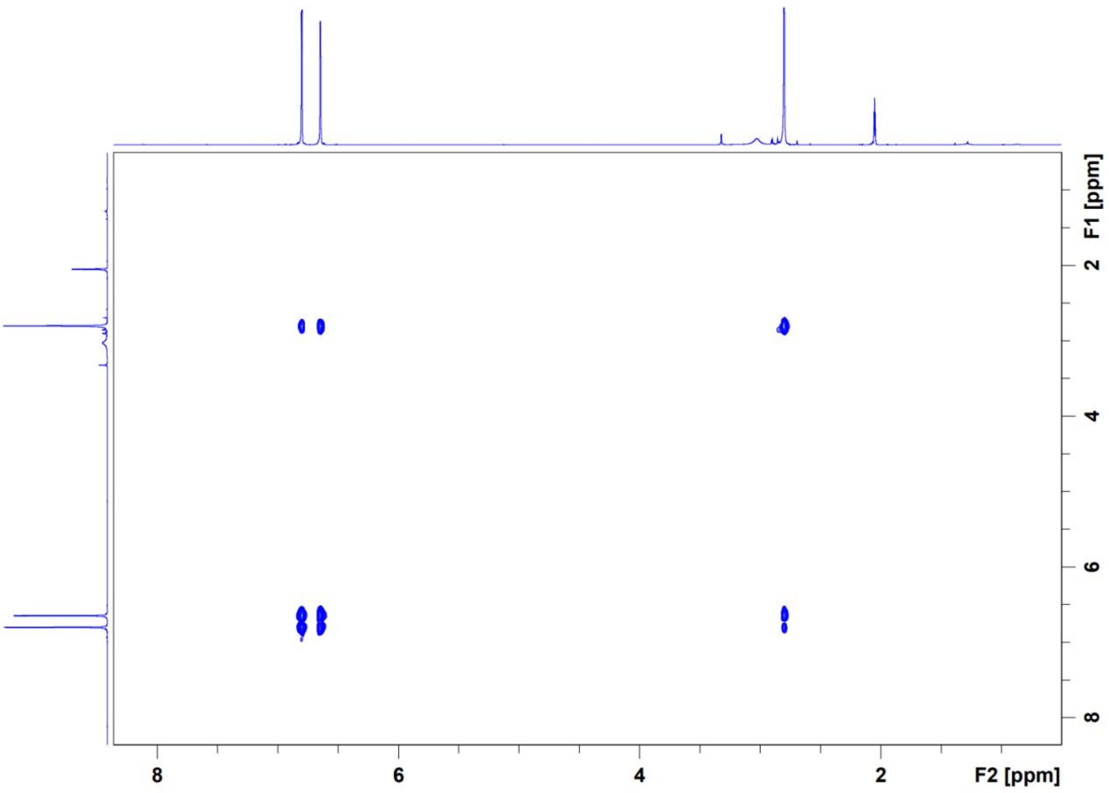

Figure S24 ^{1}H-^{1}H COSY spectrum of 3,7-dihydroxy-1,9-dimethyldibenzofuran (**3**) (Acetone-d_6)

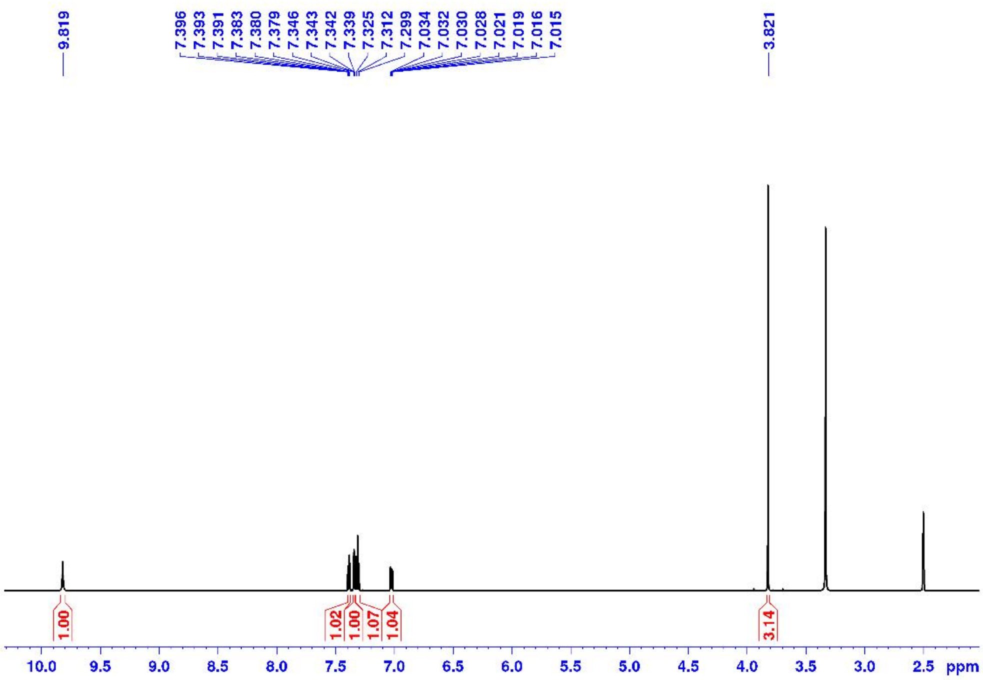

Figure S25 ^{1}H NMR spectrum of Methyl 3-hydroxybenzoate (**4**) (DMSO-d_6, 600 MHz)

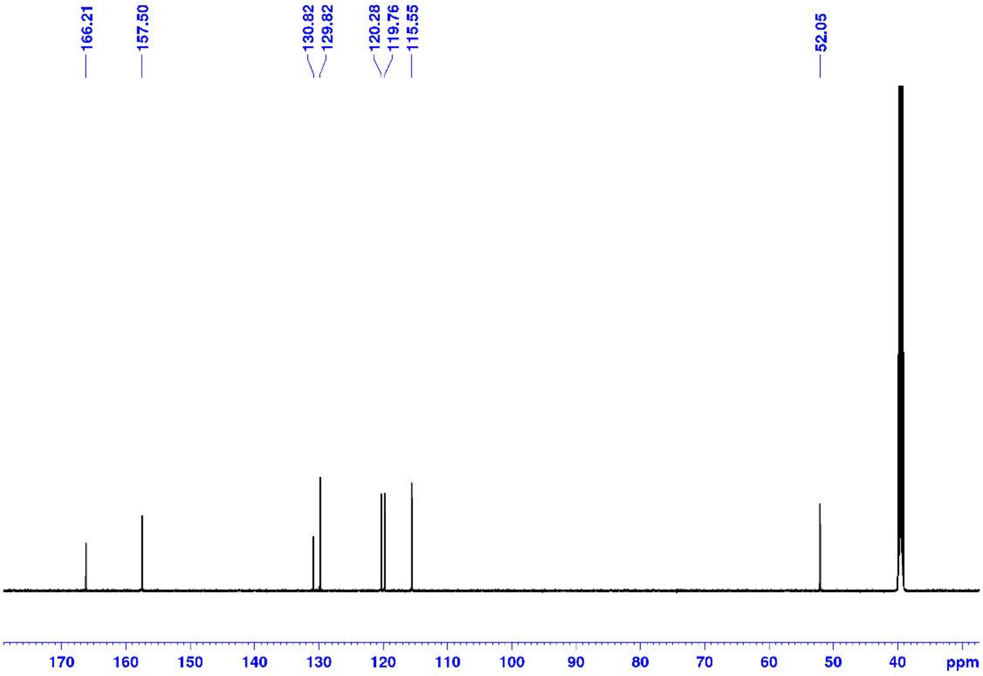

Figure S26 ^{13}C NMR spectrum of Methyl 3-hydroxybenzoate (**4**) (DMSO-d_6, 151 MHz)

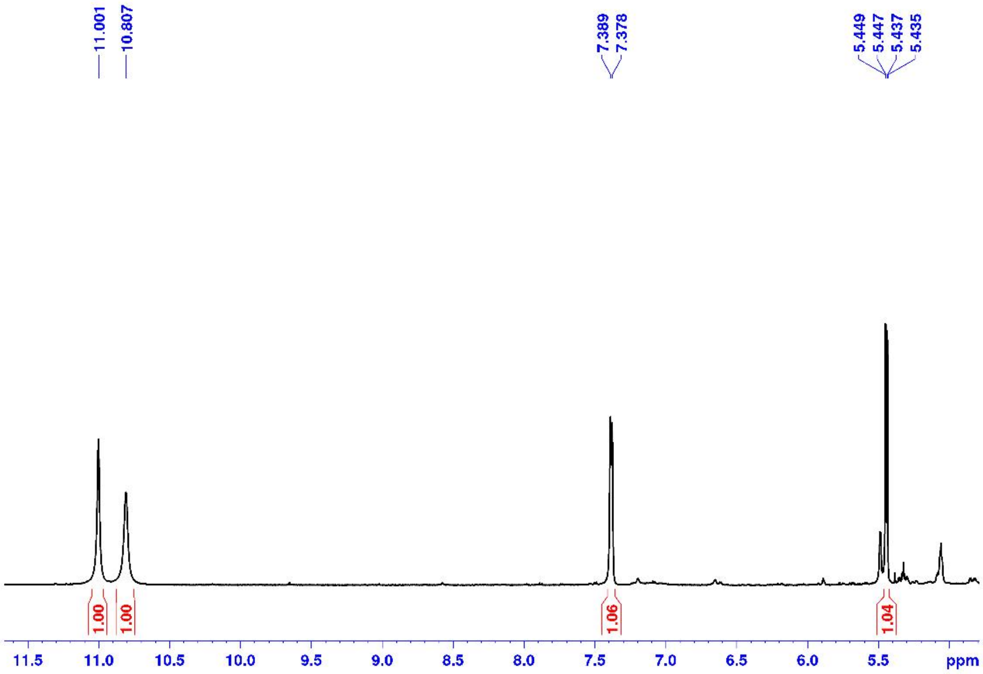

Figure S27 ^{1}H NMR spectrum of Uracil (**5**) (DMSO-d_6, 600 MHz)

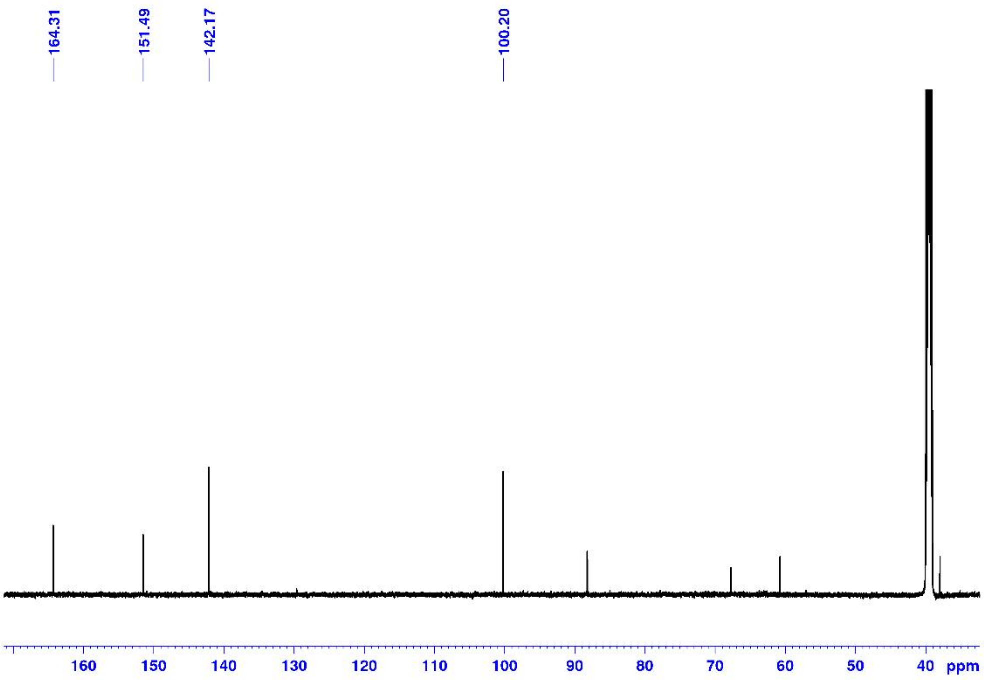

Figure S28 ^{13}C NMR spectrum of Uracil (**5**) (DMSO-d_6, 151 MHz)
